# Diagnostic blood RNA profiles for human acute spinal cord injury

**DOI:** 10.1084/jem.20201795

**Published:** 2021-01-29

**Authors:** Nikos Kyritsis, Abel Torres-Espín, Patrick G. Schupp, J. Russell Huie, Austin Chou, Xuan Duong-Fernandez, Leigh H. Thomas, Rachel E. Tsolinas, Debra D. Hemmerle, Lisa U. Pascual, Vineeta Singh, Jonathan Z. Pan, Jason F. Talbott, William D. Whetstone, John F. Burke, Anthony M. DiGiorgio, Philip R. Weinstein, Geoffrey T. Manley, Sanjay S. Dhall, Adam R. Ferguson, Michael C. Oldham, Jacqueline C. Bresnahan, Michael S. Beattie

**Affiliations:** 1 Weill Institute for Neurosciences, Brain and Spinal Injury Center, University of California, San Francisco, San Francisco, CA; 2 Department of Neurological Surgery, University of California, San Francisco, San Francisco, CA; 3 Zuckerberg San Francisco General Hospital and Trauma Center, San Francisco, CA; 4 Brain Tumor Center, University of California, San Francisco, San Francisco, CA; 5 Orthopaedic Trauma Institute, Department of Orthopaedic Surgery, University of California, San Francisco, San Francisco, CA; 6 Department of Neurology, University of California, San Francisco, San Francisco, CA; 7 Department of Anesthesia and Perioperative Care, University of California, San Francisco, San Francisco, CA; 8 Department of Radiology and Biomedical Imaging, University of California, San Francisco, San Francisco, CA; 9 Department of Emergency Medicine, University of California, San Francisco, San Francisco, CA; 10 Weill Institute for Neurosciences, Institute for Neurodegenerative Diseases, Spine Center, University of California, San Francisco, San Francisco, CA; 11 San Francisco Veterans Affairs Healthcare System, San Francisco, CA

## Abstract

Diagnosis of spinal cord injury (SCI) severity at the ultra-acute stage is of great importance for emergency clinical care of patients as well as for potential enrollment into clinical trials. The lack of a diagnostic biomarker for SCI has played a major role in the poor results of clinical trials. We analyzed global gene expression in peripheral white blood cells during the acute injury phase and identified 197 genes whose expression changed after SCI compared with healthy and trauma controls and in direct relation to SCI severity. Unsupervised coexpression network analysis identified several gene modules that predicted injury severity (AIS grades) with an overall accuracy of 72.7% and included signatures of immune cell subtypes. Specifically, for complete SCIs (AIS A), ROC analysis showed impressive specificity and sensitivity (AUC: 0.865). Similar precision was also shown for AIS D SCIs (AUC: 0.938). Our findings indicate that global transcriptomic changes in peripheral blood cells have diagnostic and potentially prognostic value for SCI severity.

## Teaser

This study demonstrates how gene expression profiles of white blood cells in acute spinal cord injury patients can be utilized for the diagnosis of the injury severity and the neurological level of injury.

## Introduction

Precision or “personalized” medicine promises to optimize individualized treatment options based on demographic and genetic characteristics as well as the specific biological features of the presenting disorder. Cancer therapy has already benefited greatly from this approach (*Nature Cancer, 2020*), where blood- and tissue-based bioassays are now routinely used for treatment planning (Lim et al., 2019). Here, we present a strategy for extending this approach to the diagnosis and treatment of human spinal cord injury (SCI), a devastating and heretofore intractable condition characterized by injury heterogeneity and highly variable outcomes. Currently, SCI prognostics are based principally on acute evaluation of neurological status using sensory and motor exams, including the American Spinal Injury Association (ASIA) grading system (Roberts et al., 2017), which in the acute phase can be unstable and difficult to obtain, especially when patients are unresponsive or obtunded (Elizei and Kwon, 2017). Magnetic resonance imaging provides invaluable information on severity and spinal cord level of injury but is not always available and may be contraindicated for certain patients, e.g., those injuries with penetrating metal.

The first attempts to discover SCI biomarkers of initial injury severity and long-term outcomes date back four decades (Norris-Baker et al., 1981). Progress has been slow due at least in part to the diffuse regional presentation of acute SCIs and the difficulty of obtaining ultra-acute samples and patient data. Most attempts have used proteomics to identify serum and cerebrospinal fluid (CSF) biomarkers associated with injury severity. This approach depends upon measuring proteins associated with central nervous system (CNS) damage (e.g., glial fibrillary acidic protein [GFAP], neurofilament protein) released into the bloodstream, or on the peripheral cytokine response to CNS injury–induced chemokines (Hulme et al., 2017; Kwon et al., 2017). Recent work shows promise, with several target molecules providing some useful predictive value (Kwon et al., 2019; Leister et al., 2020; Tigchelaar et al., 2019); however, these circulating protein (and recently, RNA) markers are difficult to measure and subject to degradation. An alternative approach is to consider that circulating immune cells represent “sensors” of CNS injury–induced molecules, and that white blood cell (WBC) transcriptomic changes provide a readout of the complex peripheral immune response to the totality of signals associated with SCI over time. A recent study of WBCs in people with chronic SCI, for example, found reduced expression of genes associated with natural killer (NK) cell activity and increased expression of Toll-like receptor and inflammatory cytokine genes (Herman et al., 2018). These preliminary findings, although limited by a small number of cases, are consistent with observations that people with chronic SCI have suppressed immune responses and are more susceptible to infections (Prüss et al., 2017).

TRACK-SCI (Transforming Research and Clinical Knowledge-SCI) is a multicenter prospective clinical study focused on acute critical care variables (e.g., magnetic resonance imaging, multiple physiological variables, time to surgery) and blood transcriptomics as indices of severity and predictors of outcome (Dhall et al., 2018; Ehsanian et al., 2020; McCoy et al., 2019; Talbott et al., 2015). SCI has a profound impact on circulating WBCs, inducing peripheral inflammation and WBC phenotypic changes in a dynamic cascade that is likely to reflect the biological features of the evolving CNS lesion (Bloom et al., 2020; Marbourg et al., 2017). TRACK-SCI provides a large WBC transcriptomic dataset that will be useful for developing RNA-based blood biomarkers of injury and recovery that can be related to patient characteristics and multivariate outcomes. Using advanced analytic methods, these blood biomarkers, along with other critical care variables, may be instrumental in the development of predictive algorithms for acute SCI treatment planning, to stratify patients for clinical trials, and to predict long-term outcomes.

We are using deep RNA sequencing (RNA-seq) and advanced analytics to develop a blood RNA biomarker profile for acute SCI. Here, we report that early WBC transcriptomic signatures alone can accurately predict injury severity on the ASIA Impairment Scale (AIS). Further, these signatures provide novel biological data that should be useful in understanding mechanisms of injury and repair. Our findings provide proof of concept for the development of an accurate blood RNA biomarker of acute SCI severity. These data provide a strong rationale for expanding this work to include longitudinal multivariate analysis of gene expression patterns across injury severities, individual patient characteristics, and time, in order to provide a comprehensive description of evolving WBC gene expression patterns and their relationships to long-term outcomes.

## Results and discussion

**Figure S1.**
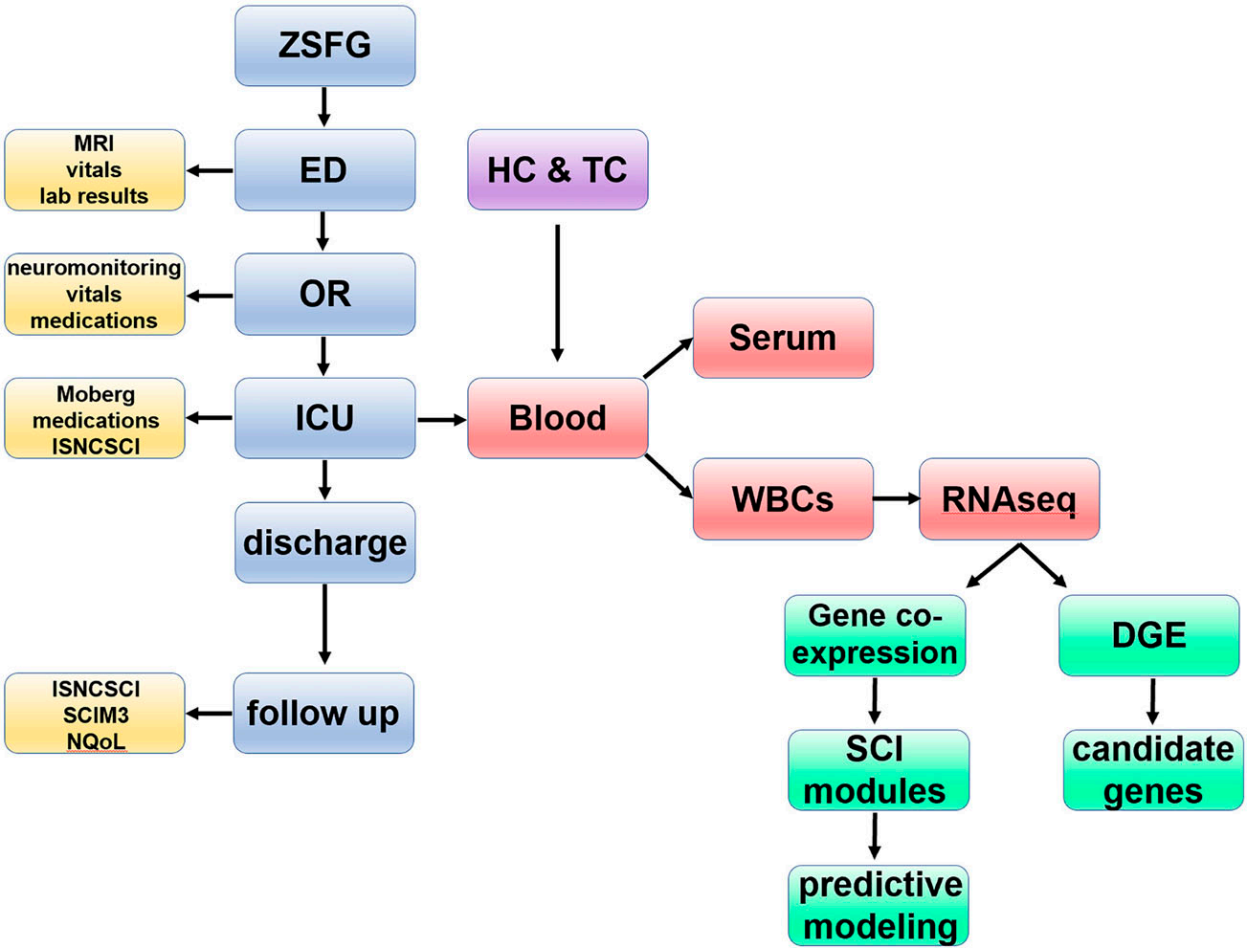
**Flowchart of patient enrollment, data acquisition, and analytic pipeline.** In TRACK-SCI, as soon as a confirmed SCI patient is admitted and consents to participate in the study, our team collects clinical data during all stages of the hospital stay and at 3, 6, and 12 mo after injury (in total >22,000 data points for each patient). Blood is also collected as early as possible after hospital admission (day 0) and at days 1, 2, 3, and 5 as well as at 6 and 12 mo after injury. After the blood draw, WBCs are isolated, and RNA is extracted for RNA-seq. The RNA-seq data from SCI patients along with RNA-seq data from HCs and TCs are analyzed using both supervised and unsupervised methods with the goal of creating a predictive model for injury severity. ED, emergency department; ICU, intensive care unit; Moberg, data collected from the Moberg ICU device; MRI, magnetic resonance imaging; NQoL, quality of life in neurological diseases; OR, operating room; ZSFG, Zuckerberg San Francisco General Hospital and Trauma Center.

TRACK-SCI protocols for patient enrollment and data collection have been described recently (Tsolinas et al., 2020). Patients admitted to the emergency department at the Zuckerberg San Francisco General Hospital and Trauma Center are recruited to the study and consented as soon as possible after admission. The TRACK-SCI protocol includes rapid preoperative imaging, emergent transfer to the operating room for decompression surgery as indicated, and immediate blood collection and processing, followed by high-density intensive care unit monitoring of vitals and daily sensorimotor and International Standards for the Neurological Classification of Spinal Cord Injury (ISNCSCI) exams (Fig. S1). To date, 179 participants with SCI have been enrolled. The current report is based on deep sequencing of RNA from acute blood samples from 38 subjects with SCI, 10 healthy uninjured controls (HC), and 10 trauma controls with non-CNS injuries (TC).

**Figure 1.**
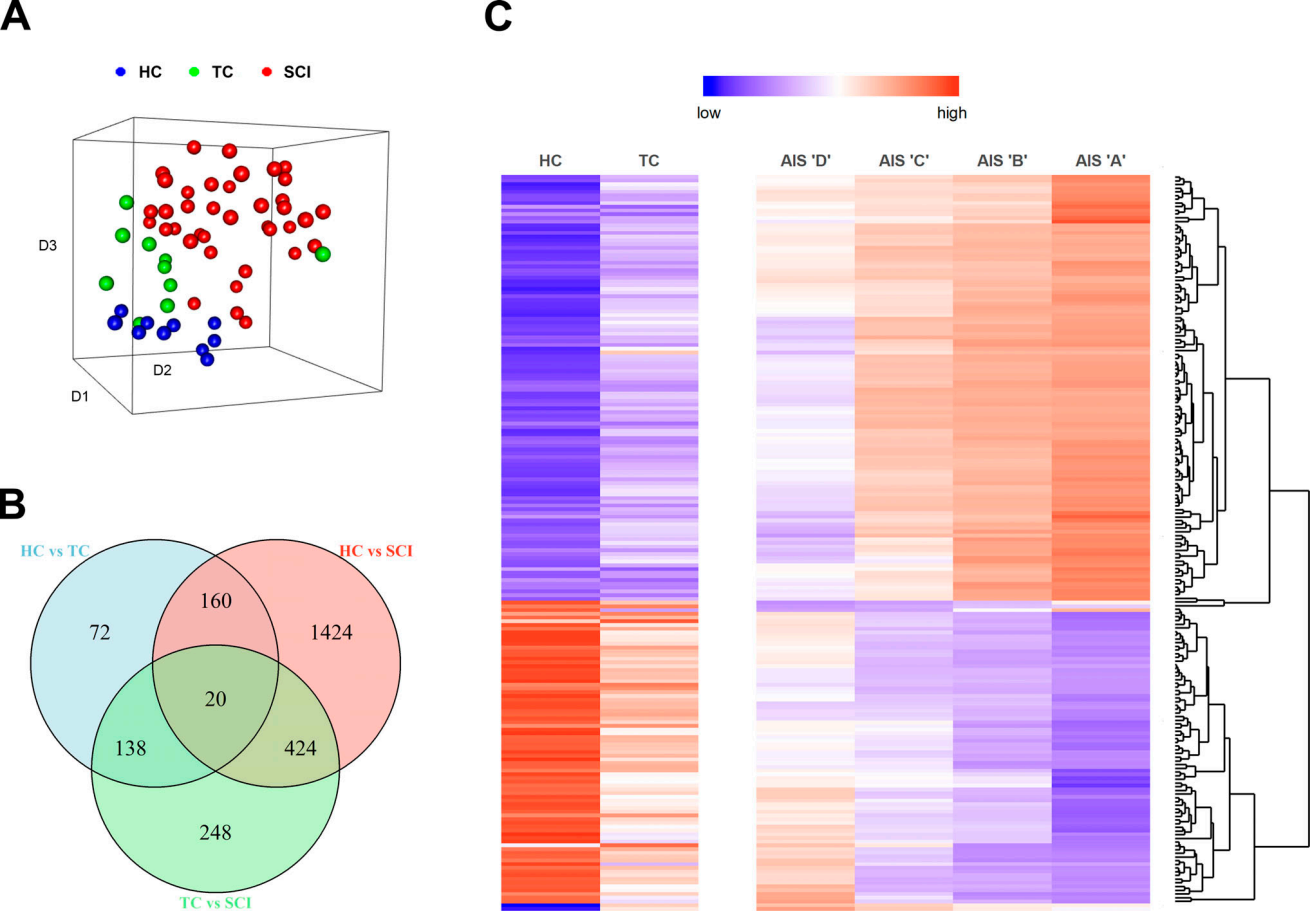
**SCI induces transcriptomic changes in WBCs compared with healthy and non-CNS TCs. (A)** three-dimensional T-distributed stochastic neighbor embedding plot. Each point on the plot represents one patient. The gene expression values of 17,500 transcripts were used in a principal component analysis, and the components that account for 90% of the variance were collapsed in the three dimensions of the T-distributed stochastic neighbor embedding plot. The three groups (HC, TC, and SCI) occupy different locations in the three-dimensional space, indicating that the transcriptomic signature alone is sufficient to separate them (HC = 10, TC = 10, SCI = 38). **(B)** Differential gene expression analysis. The Venn diagram shows the intersection between differentially expressed genes for all three comparisons between HC, TC, and SCI patients (fold-change >2, adjusted P value < 0.05). **(C)** From the Venn diagram, we selected the genes that are only significantly changed after SCI and not in the event of trauma (1,424 + 424 + 248 = 2,096). Out of those 2,096 genes, 197 exhibit changes according to the AIS grade. The heatmap shows the expression pattern of these 197 genes. The genes were selected based on their expression only in the SCI group, but the heatmap includes the levels of these genes also in HCs and TCs. The upper part shows 117 genes whose expression increases as SCI severity increases, and the bottom part shows 80 genes whose expression decreases as SCI severity increases (HC = 10, TC = 10, AIS D = 11, C = 6, B = 4, A = 12; AIS grade evaluated between 3 and 10 d after SCI, and five patients did not receive an examination during that timeframe).

**Figure S2.**
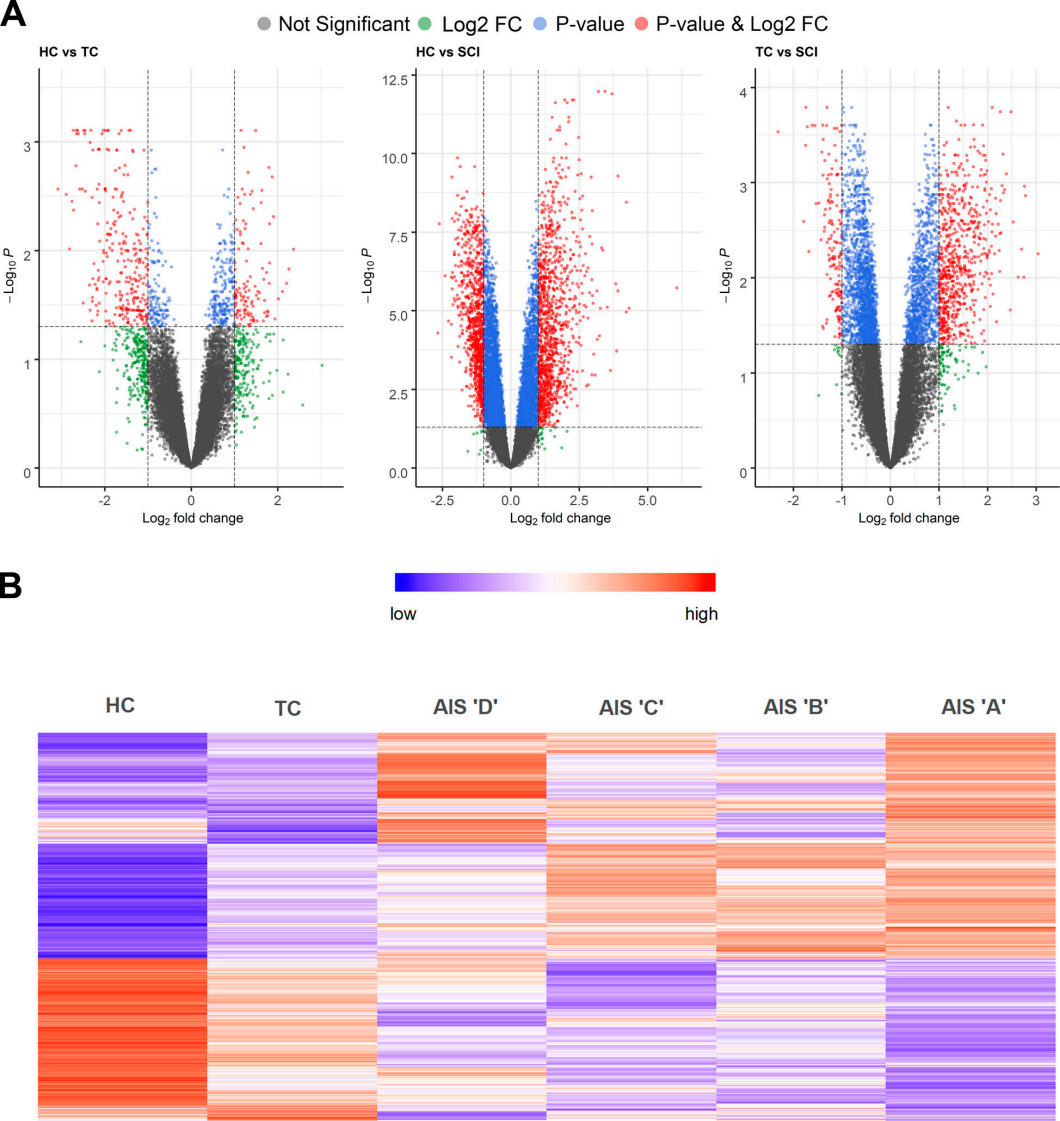
**Differential gene expression analysis of SCI patients vs. healthy and TCs reveals many genes induced specifically upon SCI. (A)** Volcano plots of the three comparisons between HCs, TCs, and SCI patients. **(B)** Heatmap of the 2,096 differentially expressed genes after SCI but not trauma (fold-change [FC] >2, adjusted P value <0.05; HC = 10, TC = 10, AIS D = 11, C = 6, B = 4, A = 12).

**Figure S3.**
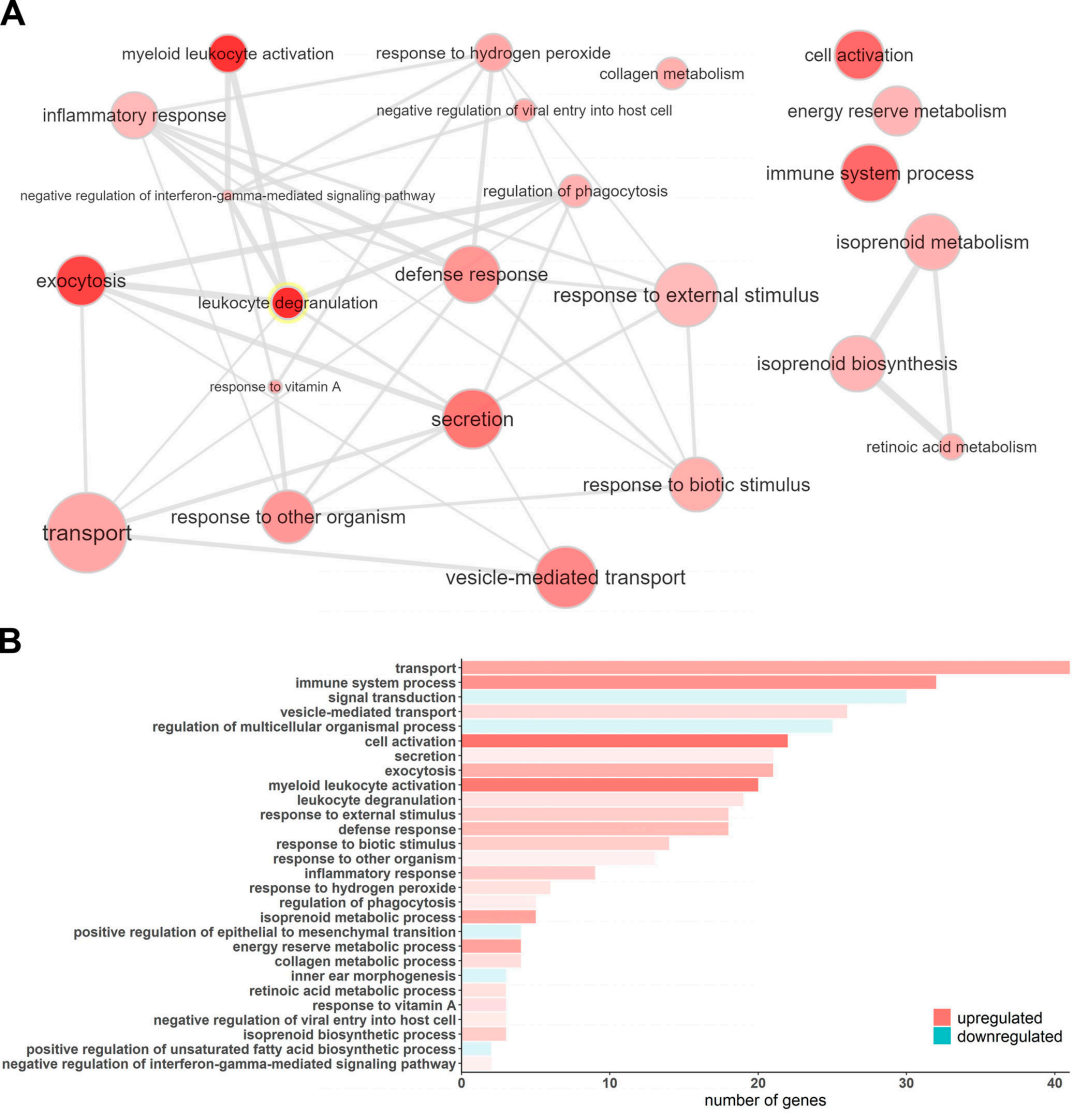
**GO enrichment analysis of the SCI severity–dependent genes suggests an important role of inflammation and cellular transport and localization in classifying SCI patients. (A)** Visualization of the enriched GOs of the genes that increase their expression as the AIS grade increases. The bubble color shading indicates the P values (stronger shading = lower P value) and the bubble size the frequency of the GO in the underlying GO Annotation database. The lines link highly similar GO terms and the width of the line indicates the degree of similarity. **(B)** The bar plot shows the number of differentially expressed genes in each one of the significant GO terms. The shade of each bar indicates the P value (stronger shading = lower P value).

We isolated 4 ml of peripheral blood from 38 enrolled patients within a few hours (Fig. S1 and Table S1) after SCI. The blood was immediately processed, and total RNA was isolated from WBCs. The same procedure was followed for 10 TCs and 10 HCs. After RNA-seq (the raw sequencing data and the normalized expression matrix can be found at the Gene Expression Omnibus database with accession no. GSE151371), raw counts were produced and normalized, and a T-distributed stochastic neighbor embedding plot was created using the principal components responsible for 90% of the variance (Fig. 1 A). The three groups are clearly separated based on their transcriptomic status at the time of the blood draw. These results support our hypothesis that the transcriptomes of WBCs contain valuable information about the pathophysiological status of the patients and warrant a more sophisticated and deeper analysis to reveal more details. Next, we performed differential gene expression analysis among the three groups, revealing 2,096 genes that were significantly altered (greater than twofold change; adjusted P value <0.05) only in the SCI population (Figs. 1 B and S2). We then queried how many of those genes display an expression pattern that follows the injury severity levels of the AIS grade. Among the 2,096 genes that were differentially expressed after SCI, 197 of them showed directional expression patterns with SCI severity (Table S2). 117 of them increased their expression with injury severity, and 80 decreased their expression with injury severity (Fig. 1 C). Gene ontology (GO) enrichment analysis showed that processes involved in the immune response and cellular secretion and localization were the most highly enriched, as expected (Fig. S3).

**Figure 2.**
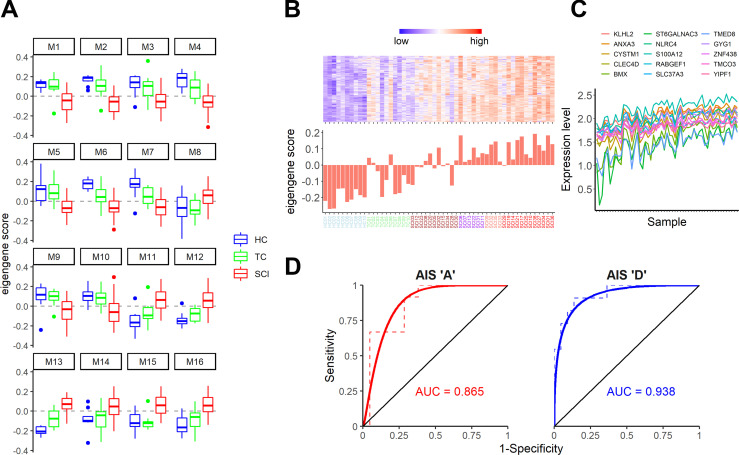
**Gene coexpression network analysis reveals transcriptional modules in peripheral WBCs that predict SCI severity. (A)** Analysis of module eigengene (PC1) scores by patient cohort reveals 16 SCI-specific gene coexpression modules following unsupervised gene coexpression network analysis (one-way ANOVA, adjusted P value <0.05, Tukey’s P value < 0.05 for each comparison). Some modules (e.g., M4) display a gradual change in gene expression, whereas in others (e.g., M1, M5), HCs and TCs are very similar to each other but different from SCIs. *n* = 10 for HCs and TCs and 38 for SCIs. **(B and C)** The M13 module has the highest correlation to SCI severity (Spearman ρ = 0.82). In B is the heatmap of the top-seeded genes for this module (top), and the eigengene score for each one of the patients and controls (bottom). The graph in C shows the expression levels of the top 15 genes of the M13 module across all 58 samples. As expected from the analysis, these top genes of the module exhibit a strong coexpression pattern. **(D)** Receiver operating characteristic plots for the AIS A against the remaining SCIs (left) and the AIS D against the remaining SCIs (right). These plots show the strong predictive ability of our model for SCI patients with AIS A and D. The area under the curve (AUC) is 0.865 for A and 0.938 for D. *n* = 12 A vs. 21 SCIs and 11 D vs. 22 SCIs (color scheme in x-axis labels in B is as follows: blue = HC, green = TC, brown = AIS D, purple = AIS C, salmon = AIS B, and red = AIS A).

Variation in the abundance of cell types and cell states in heterogeneous samples has been shown to drive covariation of gene expression patterns in a variety of biological systems (Langfelder and Horvath, 2007; Oldham et al., 2006; Oldham et al., 2008; Oldham et al., 2012). Therefore, we performed unsupervised gene coexpression network analysis (Kelley et al., 2018) and identified 16 modules (arbitrarily designated M1–M16) for which SCI patients’ combined expression was significantly different from both TCs and HCs (Fig. 2 A). These modules represent coherent transcriptomic signatures in WBCs that covary specifically as a result of SCI and are therefore targets for biomarker generation as well as potential indicators of underlying pathology and recovery. Among these, M13 exhibited the highest correlation with AIS grade (Spearman ρ = 0.82; P value = 1.56 × 10^−14^). Fig. 2, B and C, shows the details of top gene coexpression patterns for this module.

**Figure 3.**
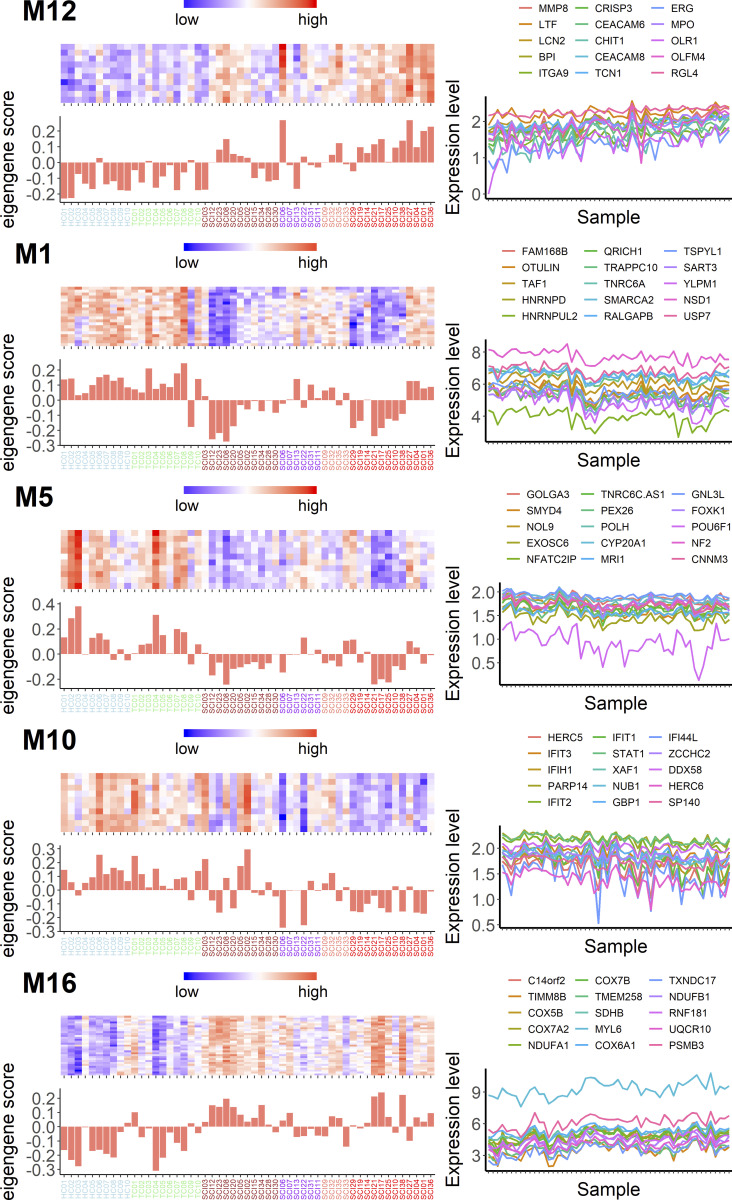
**Multinomial logistic regression identifies specific gene modules with the capacity to accurately predict AIS A and AIS D SCI patients.** For AIS A SCI patients, one gene module (M12) is sufficient to predict the injury class with 83.3% accuracy. Interestingly, five gene modules (M13 in Fig. 2, B and C; M1, M5, M10, and M16) are required to predict AIS D SCI patients with an impressive 90.9% accuracy. For each one of the modules in this figure, on the left is a heatmap with the top-seeded genes for the module and the eigengene score for each patient (and control); on the right are the expression patterns (in arbitrary units) of the top 15 genes with the highest correlation to each module eigengene (color scheme in x-axis labels is as follows: blue = HC, green = TC, brown = AIS D, purple = AIS C, salmon = AIS B, and red = AIS A).

Next, we wanted to determine whether any of these 16 modules, alone or in combination, could predict the initial SCI severity as indicated by the AIS grade assigned between days 3 and 10 after SCI. We therefore performed multinomial logistic regression with least absolute shrinkage and selection operator regularization (Friedman et al., 2010) to predict AIS grade using all 16 module eigengenes. We identified one gene module (M12; Fig. 3) that predicted AIS A patients with 83.3% accuracy and a combination of five modules (M1, M5, M10, M13, and M16) that predicted AIS D patients with 90.9% accuracy (Fig. 3, Table S3, and Table S4). Our cohort included too few patients with B or C classifications (*n* = 4 and 6, respectively) to provide useful predictors of these grades (Table S5). Overall, our model shows 72.7% accuracy (P = 2.35 × 10^−5^). We proceeded to test the diagnostic value of the identified modules to detect AIS A and D patients in our cohort using a receiver operating characteristic (ROC) analysis. The areas under the curve for AIS A patients and AIS D patients were 0.865 and 0.938, respectively, confirming that our model can predict these two injury severities with high sensitivity and specificity, despite small sample sizes (Fig. 2 D).

**Figure 4.**
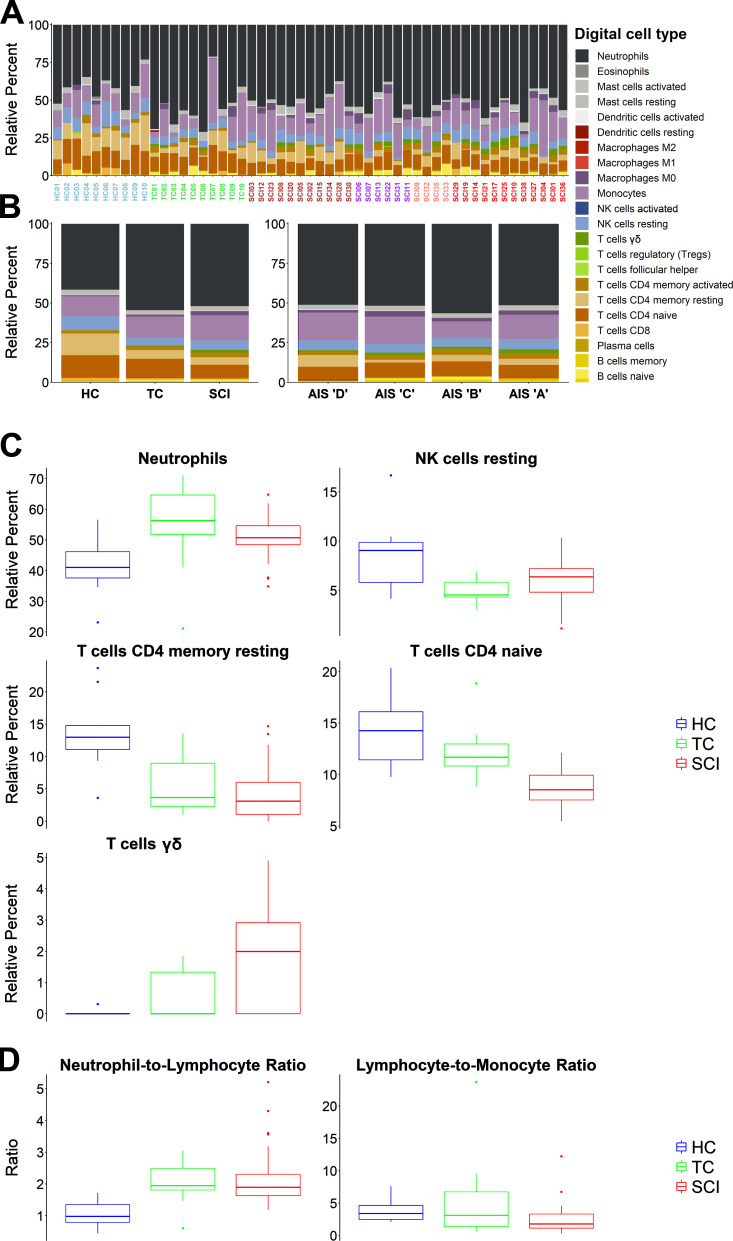
**Digital cytometry using CIBERSORTx measures relative abundance of 22 distinct leukocyte subtypes in SCI and control patients.** We used a recently created machine learning algorithm (CIBERSORTx) that uses deconvolution methods to infer cell type proportions based only on gene expression patterns. We cross-referenced the transcriptomes of all SCI patients and controls with the leukocyte gene signature matrix (LM22; Newman et al., 2015) and estimated relative abundances for 22 leukocyte subtypes. **(A)** The stacked bar plots show the relative abundance of the 22 digital cell types for each of the SCI patients and controls. **(B)** Left shows group averages, and right shows AIS grade averages. **(C)** One-way ANOVA for each digital cell type showed statistically significant differences for neutrophils, resting NK cells, CD4 resting T cells, CD4 naive T cells, and γδ T cells with adjusted P values <0.05. Tukey’s test showed that for CD4 naive and γδ T cells, the SCI group is significantly different from both control groups. No statistically significant difference was identified among AIS grades. **(D)** The neutrophil-to-lymphocyte and lymphocyte-to-monocyte ratios (calculated from the CIBERSORTx data) show differences between HCs, TCs, and SCIs (one-way ANOVA, P value < 0.05; color scheme in x-axis labels in A are as follows: blue = HC, green = TC, brown = AIS D, purple = AIS C, salmon = AIS B, and red = AIS A).

To determine whether cellular composition varied among WBCs from our sample cohorts, we performed cell-type deconvolution using CIBERSORTx (Newman et al., 2015; Newman et al., 2019), which infers cell-type proportions in bulk tissues from global gene expression patterns. Applying this method to our samples revealed the estimated relative proportions of 22 leukocyte subtypes. These “digital cell types” were then compared across groups (HC, TC, and SCI) and AIS grade levels (Fig. 4, A and B). Five cell types (neutrophils, resting NK cells, resting CD4, naive CD4, and γδ T cells) exhibited significantly different proportions among the three groups (Fig. 4 C), but none of these were significantly different between AIS grades. We also used these inferred proportions to calculate leukocyte ratios, which have been proven clinically useful in multiple diseases and traumas (Choi et al., 2020; Quan et al., 2020; Zhao et al., 2020). The neutrophil-to-lymphocyte and the lymphocyte-to-monocyte ratios were both significantly different between the three groups (one-way ANOVA P values 0.0009 and 0.0273, respectively; Fig. 4 D) but did not differ across AIS grades. Thus, changes in leukocyte ratios were indicators of the presence of SCI, but by themselves were not sufficient to predict injury severity.

To clarify which cell types contributed to the coexpression modules that predict SCI severity, we cross-referenced module composition with cell type–specific gene sets from published RNA-seq datasets (Newman et al., 2015; Watkins et al., 2009). The M12 module, which predicted AIS A injury severity, was significantly enriched with genes expressed by resting NK cells, mast cells, and CD66^+^ granulocytes (Fisher’s exact test P values of 2.9 × 10^−7^, 5.19 × 10^−7^, and 7.81 × 10^−6^, respectively). The M13 module, which predicted AIS D severity, was also significantly enriched with CD66^+^ granulocytes (P = 1.45 × 10^−8^). These data suggest that expression changes in WBCs associated with SCI can be further subdivided into specific contributions from distinct cell types, which could lead to refined assays and predictors.

**Figure 5.**
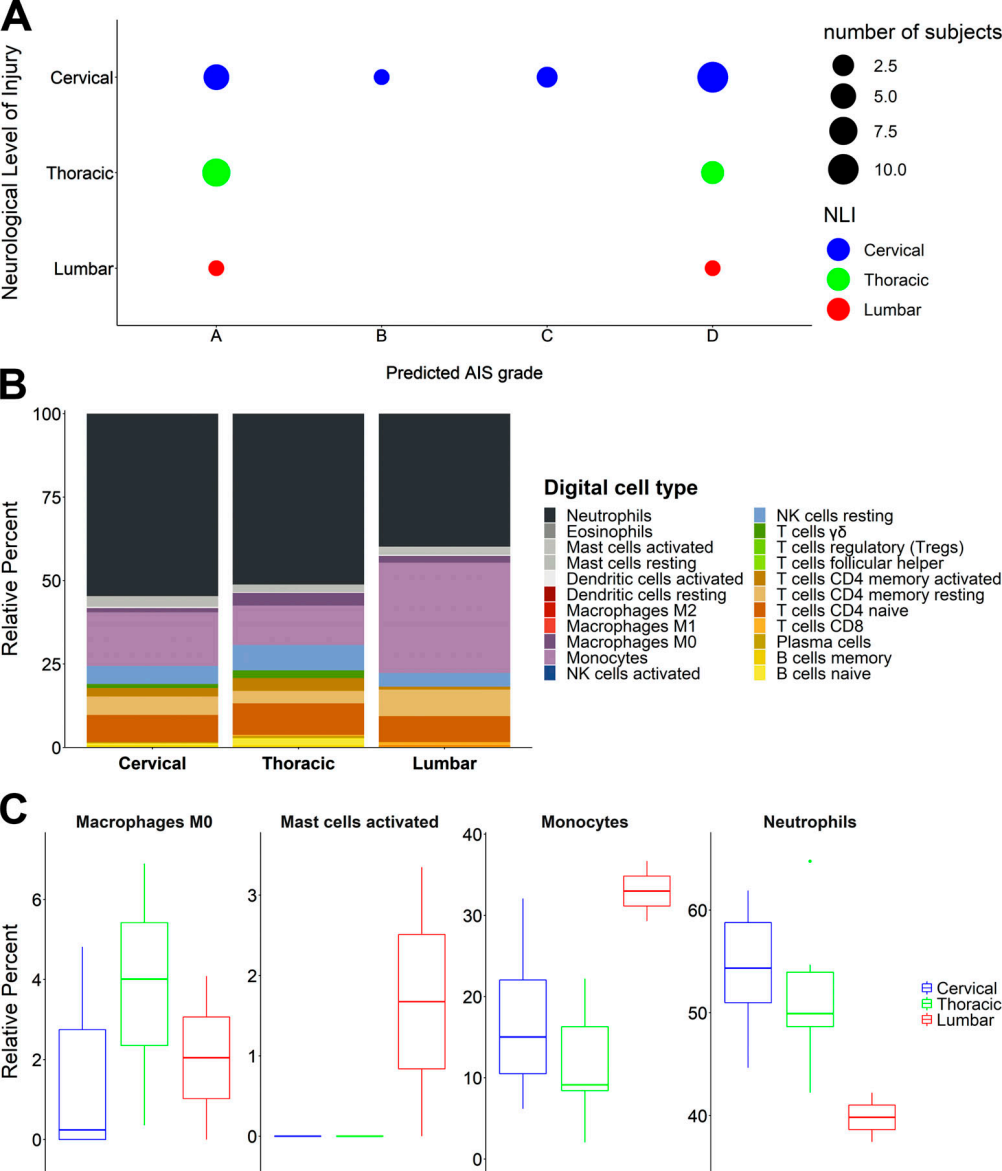
**NLI is correlated with a differential immunological response after SCI. (A)** The distribution of the NLI levels across the predicted AIS grades does not show a pattern that would suggest a strong effect on the predictability of the model. **(B)** CIBERSORTx data aggregated per NLI. The stacked bar plots show the differential immune response for each NLI level. **(C)** One-way ANOVA for each digital cell type from B showed that several cell types displayed a statistically significant differential response per NLI. The four cell types presented here are the ones remaining significant (P < 0.05) after Benjamini–Hochberg multiple testing correction (cervical = 18, thoracic = 10, lumbar = 2).

AIS grades of severity by themselves do not indicate neurological levels of injury (NLI), although NLIs are clearly critical for determining functional outcomes. Therefore, we wanted to test whether the NLI in our SCI cohort could have affected the predictability of our model. This is especially important taking into consideration the imbalance of our cohort in the NLI (>50% are in the cervical region, <10% lumbar; Table S5). Although sample size restrictions do not allow us to evaluate the NLI as a confounding variable, we sought to analyze their distribution against the predicted AIS grades. The results showed that no NLI level is significantly responsible for the predicted AIS grades and that they are relatively evenly spread across them (Fig. 5 A). This result does not mean that the NLI could not be a confounding variable, but it rather shows that our predictive model is not affected by the cohort NLI imbalance.

While we showed that the NLI probably does not affect the predictability of our model, that does not preclude the possibility that WBC transcriptomes may be affected by it. Indeed, there is strong evidence in the literature suggesting that different immunological responses can be induced in cervical vs. thoracic or lumbar SCI (Hong et al., 2018). Using our CIBERSORTx estimates, we were able to verify the differential digital immunological phenotypes resulting from SCI in the cervical (*n* = 18), thoracic (*n* = 10), and lumbar (*n* = 2) regions (Fig. 5 B). In particular, we could see that the proportions of neutrophils, monocytes, M0 macrophages, and activated mast cells are significantly different across the three NLI levels (Fig. 5 C). Continuing to explore the relationship between NLI and WBC transcriptomes, we sought to discover if the 16 SCI-induced modules we identified (Fig. 2 A) could be used to predict whether the SCI is in the cervical region. Again, using logistic regression with least absolute shrinkage and selection operator regularization, we identified a combination of three modules (M10, M12, and M16) that could predict whether the SCI is cervical with 73.6% accuracy (P value = 0.04352; Table S6 and Table S7). These data offer a first glimpse of the possibilities emerging from our analytical pipeline. With a larger sample size, it is likely that we will not only identify diagnostic severity biomarkers but also get insights on specific anatomical and biological characteristics of the injury in addition to the peripheral immune molecular response.

Early biomarkers of SCI could enable more efficient and personalized clinical treatments, as well as better stratification of patients for clinical trials, since early clinical evaluations often lack reliability (Hulme et al., 2017; Kwon et al., 2019). In contrast to existing biomarkers that require rapid access to complex machinery (e.g., magnetic resonance scanning), fluid biomarkers are more accessible and can provide novel insights about systemic responses to SCI. Although previous studies have proposed large structural proteins in blood serum/plasma and CSF as fluid biomarkers of SCI (Kwon et al., 2017; Leister et al., 2020), these proteins are susceptible to proteases and degrade rapidly, rendering measurements of concentration variable- and time-sensitive. More recent studies of circulating microRNAs as biomarkers are promising since microRNAs are not as sensitive to degradation due to their small size (22 nt) and the fact that most are protected inside exosomes (Tigchelaar et al., 2019; Tigchelaar et al., 2017).

Our approach differs substantially from previous efforts to identify fluid biomarkers of SCI. First, we have analyzed potential biomarkers that are “safely housed” in their cell of origin at the time of collection, as opposed to free-floating molecules in CSF or blood. Second, instead of preselecting candidate biomarkers in advance, we have performed an unbiased high-throughput analysis of 17,500 transcripts from WBCs isolated from each patient. This high-dimensional readout of the immune response during the acute phase of the injury provides important information about how the periphery affects the progress of the central lesion and may lead to new hypotheses and targets for intervention. Our results indicate that global gene expression patterns in WBCs can distinguish SCI patients from healthy and non-CNS TCs. Moreover, when overlaying these gene expression patterns with the widely used AIS injury grade classification system, we identified 197 genes whose expression levels changed with increasing injury severity and, upon confirmation in larger patient cohorts, may serve as novel fluid biomarkers of SCI. The list of these 197 genes (Table S2) includes a number of genes that seem appropriate, as well as some whose functions are yet to be delineated, which might provide clues to new therapeutic targets. For example, metalloproteinase-8 (*MMP8*), which is the top-seeded gene in module M12 (module predicting AIS A), haptoglobin (*HP*), and kininogen-1 (*KNG1*) have each been proposed previously as potential biomarkers for SCI, at the protein level in either blood plasma/serum or CSF (Light et al., 2012; Lubieniecka et al., 2011; Sengupta et al., 2014). The fact that we identify them at the mRNA level in WBCs and they seem to be correlated with SCI severity is very promising and provides converging evidence for the potential importance of these genes as encoding transcript and protein indicators of injury. Due to the high throughput of our approach, we have dozens more genes to screen that have never been reported as potential SCI biomarkers. For example, our analysis identified *MCEMP1* (mast cell–expressed membrane protein 1) as a candidate biomarker of SCI severity. Interestingly, Raman et al. (2016) previously suggested that the expression level of this gene in WBCs is a biomarker for stroke prognosis. These findings may indicate a need to separate SCI-only patients from SCI patients who also suffered a concurrent traumatic brain injury or suffered from a preexisting neurological condition (Table S1), sample size permitting.

Despite the attractiveness of single-gene biomarkers, there is growing evidence (mainly from cancer research) to suggest that their performance is limited (Targonski et al., 2019). Of course, genes do not work in isolation, and there is overwhelming evidence for reproducible transcriptional covariation in blood and other tissues, suggesting a modular organization to genomic function. These modules are not always neatly captured by existing GOs, hence the need to perform unsupervised coexpression analysis. Multivariate analysis revealed 16 gene coexpression modules associated with SCI, a subset of which were able to predict AIS A and AIS D SCI patients with impressive accuracy. From a clinical perspective, this finding has the potential to add an important resource to health care providers, with the caveat that routine assays of mRNA expression patterns in the acute care setting will require the development of new technologies.

It is worth noting that the timing of the AIS grades is not always the same. For most of our patients, more than one ISNCSCI examination was performed during their hospital stay. In this study, we used the earliest AIS grade obtained between 3 and 10 d after injury. The rationale for that choice is that we wanted to stay away from the first 48 h after injury, a time point known to produce substantially unstable AIS grades due to several factors, such as spinal shock or strong sedation of the patients. Similarly, we stayed clear from the exam performed at discharge due to the wide variance at discharge times (from 4 to 56 d after injury). The range we selected for our analysis (3–10 d after injury) was the one that provided us with enough data, allowed us to avoid unnecessary technical and biological variance, and also proved to be stable across time. Only four of our patients would have a different AIS grade if we were to use their discharge exam. A future goal of our ongoing study is to predict long-term outcomes (e.g., the AIS grade at 6 and 12 mo after SCI). Currently, such an analysis is limited by sample size: Out of the 38 SCI patients, only 17 returned for their 6- and/or 12-mo evaluation, and 16 out of those 17 were classified as AIS D, so we are hampered by both sample size and low variance. We are continuing to add longitudinal data from enrolled patients. In the future, we will be able to extend our analysis in this fashion with the addition of participants both at our center and at new TRACK-SCI sites that are in the process of being added to our study. Moreover, given the well-known limitations of the AIS grade scale, additional outcome measures (e.g., upper and lower extremity motor scores and sensory scores from the ISNCSCI neurological examination) could offer more detailed insights into patients’ conditions and enable more accurate predictions. As we increase the number of enrolled patients and sequenced blood samples, such analyses will be feasible.

Although we have demonstrated proof of concept for our methodology in a sample of 38 SCIs, TRACK-SCI continues to enroll patients, with 179 participants to date. By combining multivariate and longitudinal analysis of WBC transcriptomes with detailed clinical information, we seek to create a novel framework for diagnosing SCI severity and predicting outcomes based on the systemic immune response. Such a framework could eventually replace the ASIA grading system or be used in combination to allow more precise clinical decisions. Additional studies with larger sample sizes will be required to validate these findings and move toward a practical RNA-based blood biomarker. Furthermore, it should be possible to determine whether diagnostic patterns reflect WBC responses to specific CNS injury–induced signals such as CNS protein products and/or signaling molecules such as chemokines. The clear differentiation between expression profiles from TCs and SCI suggests that WBC transcriptomes contain latent information specific to CNS injury, raising the possibility that WBC RNA profiles may also respond to treatments that mitigate secondary damage or are associated with recovery of function. If so, the routine analysis of RNA expression profiles in blood may provide both a practical clinical tool and a new window into the biology of human CNS injury and its treatment.

## Materials and methods

### Statistics

The differential gene expression analysis was performed using linear modeling with edgeR and the limma method in the R programming language. The genes were selected based on the P values (P < 0.05) of the Benjamini–Hochberg correction and a log2 fold change >1 or lower than −1. For eigengene module selection and CIBERSORT cell type differential analysis, one-way ANOVA was used with Tukey’s post hoc and adjusted (Benjamini–Hochberg) P values <0.05. For all the boxplots in the figures, the middle line represents the median value, the bottom and the top of the box represent the first and third quartiles of the data, the whiskers are 1.5 times the interquartile range, and the dots represent the outliers.

### TRACK-SCI patients and controls enrollment

All procedures for this study were conducted with the approval of the Human Subjects Review Boards at the University of California, San Francisco, and the U.S. Department of Defense Human Research Protection Office. All English- and non–English-speaking patients who presented to the emergency department and were diagnosed with a traumatic SCI were initially eligible for the study. Patients who were <18 yr old, in custody, prisoners, pregnant, or on medically indicated psychiatric hold were excluded. Informed consent was sought for all patients. For patients who were unable to sign for themselves due to their injury, a witness unaffiliated with the study was present throughout the consenting process and signed on the patient’s behalf. Patients incapable of consenting themselves were initially enrolled via a legally authorized representative (next of kin) or another suitable surrogate when one was available, then later approached for patient consent. Patients and surrogates had the option to participate in all or some of the following study portions: blood draws, ISNCSCI examinations, and/or follow up assessments. Patients were compensated ($50) after each time point (hospital stay, 3-mo phone call, 6-mo in-person visit, 12-mo in-person visit) for a total of $200.

Non-SCI subjects were either HCs (*n* = 10) or TCs (*n* = 10). HCs were recruited using Institutional Review Board–approved recruitment flyers posted at Zuckerberg San Francisco General Hospital and Trauma Center and from friends and family of enrolled SCI patients. Subjects contacted study coordinators, were interviewed, consented, and provided basic demographic information and biospecimens. TCs were recruited from emergency department patients with traumatic but non-CNS injuries. The same basic demographic and biospecimen data were collected for these patients as for SCI patients for comparison purposes except that a single blood sample was taken. No monetary compensation for participation was provided for the control subjects.

### Patient data collection

The foundation of the TRACK-SCI database is the National Institute of Neurological Disorders and Stroke–recommended common data elements (CDEs; Biering-Sørensen et al., 2015). Core CDEs are data elements that all SCI studies are strongly encouraged to use in collection of basic participant information. Additional measures from the International Spinal Cord Society were also used. Data collection domains include demographic, clinical, radiological, and functional outcome measures. All data collected from these CDEs were housed in a Research Electronic Data Capture (REDCap; Harris et al., 2009) database and include >21,000 data fields including additional institutional variables, calculated fields, repeated measures, date/time stamping of measures, and completion status log. Upon admission to the inpatient service, another 19,148 data fields regarding trauma characteristics, injury severity, blood pressure management, operating room procedures, interventions, hospital outcomes, and high-frequency operating room vital signs, as well as motor-sensory exams and pain questionnaires, are obtained from both paper and electronic medical records as well as participant interviews. REDCap is in full compliance with Health Insurance Portability and Accountability Act security standards for protection of personal health information. The following CDE categories comprised the demographic and clinical data domain: (1) demographics, (2) health history, (3) injury-related events, and (4) assessments and examinations. A total of 229 variables concerning patient demographics, medical history, and consent/contact information was collected through abstraction from electronic medical record systems and participant interviews.

The ISNCSCI (Maynard et al., 1997) was used to assess motor and sensory function and group patients by injury severity based on the AIS, which ranges from A (most severe—complete) to E (not impaired). ISNCSCI examinations were conducted by trained personnel who completed the ASIA International Standards Training E Program and in-person training. ISNCSCI exams were performed on all patients during the initial admission, either as part of clinical care if the treating provider completed International Standards Training E Program training, or separately for the research study if the ISNCSCI was not performed for clinical purposes. Occasionally, an ISNCSCI was not performed or not completed during the admission, usually because the patient was excessively sedated and could not participate in the exam. In the case of incomplete ISNCSCI examinations, the assessor gave an estimated AIS grade based on the collected data and the overall clinical picture of the patient.

If possible, patients completed examinations at regular intervals including admission (day 0 = 0–23 h from injury), every 24 h until post-injury day 7, discharge, 6-mo follow-up (±2 wk), and 12-mo follow-up (±2 wk). All ISNCSCI exam results were included in the REDCap database.

### Biospecimen collection

Two blood samples were collected, one 4 ml for total RNA extraction from WBCs, and the other 6 ml for serum isolation. Samples were aliquoted and frozen at −80°C within 1 h of collection. To preserve WBC concentrations, 7.2 mg K2-EDTA vacutainer tubes were used for WBC collection and subsequent RNA extraction, instead of K3-EDTA vacutainer tubes. The second blood sample was collected in 6-ml Z serum clot activator vacuette tubes for serum extraction. To prevent reduction in sample volume, externally threaded cryovials were used for serum storage. Serum was divided into multiple 500-µl aliquots for storage. An inventory system was developed to track time of collection, processing, and storage for all biospecimens.

### WBC isolation and RNA extraction

Blood was centrifuged at 1,500 rpm for 15 min at room temperature within 0–15 min from blood draw. Then, the interface layer (buffy coat) was carefully aspirated with a pipette and placed in 10 ml of 1× solution of Red Blood Cell Lysis Buffer (BioLegend) for 15 min in the dark. After the 15-min incubation, the solution was centrifuged at 1,500 rpm for 10 min. The supernatant was discarded, and the cell pellet resuspended in 1 ml of TRIZOL (Ambion) and either stored at −80°C or immediately processed for RNA extraction. Total RNA from WBCs was extracted using the TRIZOL method. The RNA yield was between 15 and 25 µg per 5 ml peripheral blood. 1 µg of the total RNA was then used for generating the Illumina cDNA library, which was used for the downstream RNA-seq.

### RNA-seq

1 µg of total RNA was used for the library synthesis. cDNA libraries were synthesized using Illumina’s TruSeq Stranded Total RNA with Ribo-Zero Globin kit. The kit depletes ribosomal RNA, which makes up more than 90% of total RNA, and globin mRNA, which is present in very high levels in blood total RNA. The libraries were quantified using a Thermo Fisher Scientific Nanodrop 2000c spectrophotometer, and their quality and average fragment size was assessed using Agilent’s DNA 1000 kit and Agilent’s 2100 Bioanalyzer. After quantification, equal amounts of 10 libraries, each one with different barcoded adapters, were pooled together to be sequenced in one lane of the Illumina’s HiSeq4000 sequencer. Based on the specifications of the HiSeq4000 and our sample pooling per lane, we aimed to get ∼40 million reads per sample, which has been shown to be sufficient to reveal the vast majority of the differentially expressed genes of a well-annotated genome (Liu et al., 2014). The sequencing output of our samples can be seen in Table S8, and the raw sequences have been deposited to Gene Expression Omnibus database with accession no. GSE151371.

### Bioinformatic analysis

Data analysis was performed in R (R Core Team, 2019; R Studio Team, 2020) using the statistical packages that are specifically mentioned below as well as the packages dplyr (Wickham et al., 2020), ggplot2 (Wickham, 2016), cowplot (Wilke, 2019), table1 (Rich, 2018), rgl (Adler and Murdoch, 2019), PCAtools (Blighe, 2019b), magick (Ooms, 2020), EnhancedVolcano (Blighe, 2019a), VennDetail (Guo and McGregor, 2019), Rtsne (Krijthe, 2015), and ggdendro (de Vries and Ripley, 2016). The raw reads of the fastq files were tested for quality control using the FastQC software (Andrews, 2010) and were then aligned to the human reference genome (hg38 from University of California, Santa Cruz) using the software TopHat2 (Kim et al., 2013). After the alignment, we used the featureCounts (Liao et al., 2014) program to summarize the gene counts, and then the programs edgeR (Robinson et al., 2010) and limma (Ritchie et al., 2015) for differential gene expression analysis through linear modeling. GO enrichment analysis was performed with the GOrilla (Eden et al., 2009) tool and the visualization of the enriched GO terms with the tool ReviGO (Supek et al., 2011).

### SCI-specific differentially expressed genes and neurological outcome

Preliminary examination of the data using network methods revealed several samples as outliers as well as library and sequencing batch effects. Using the ComBat (Johnson et al., 2007) function of the sva (Leek et al., 2012) package in R to target the library batch effect, we were able to remove both batch effects, and as a result, no sample appeared as an outlier anymore. We performed differential gene expression analysis among the three groups (HC, TC, and SCI), and after the intersection of the three comparisons (HC vs. TC, HC vs. SCI, and TC vs. SCI; fold change >2 and adjusted P value < 0.05), we selected only the genes that significantly and specifically changed their expression after SCI (*n* = 2,096). To examine the relationship between the gene expression changes and injury severity, we grouped the SCI patients based on the assigned AIS grade given after a neurological examination between days 3 and 10 at the hospital (Table S5). From the 38 SCI patients whose transcriptome was sequenced, five did not have an AIS grade during that time window and hence were removed from this part of the analysis. We then averaged the expression levels of each gene per group and queried for genes that exhibited a stepwise increased or decreased expression as the SCI severity increased. That query resulted in 197 genes shown in Fig. 1 C and Table S2.

### Gene coexpression network analysis and identification of SCI-specific gene modules

The normalized expression matrix that was generated for the differential gene expression analysis with edgeR was used as a template for the unsupervised gene coexpression network analysis (Kelley et al., 2018). We built a series of gene coexpression networks and identified one that included the gene module with the highest Spearman correlation to AIS grade. That network contained 57 gene modules. Using one-way ANOVA with Tukey’s multiple comparison correction, we identified 17 gene modules that were highly specific for SCI (significantly different from both HC and TC; adjusted P value < 0.05). One of these modules contained genes annotated to be involved in ribosomal RNA processes, which likely represented an artifact, and was eliminated from the subsequent analysis. The 16 remaining SCI-specific modules (M1–M16; Fig. 2 A) were used to create a predictive model of SCI severity using multinomial logistic regression.

### Multinomial logistic regression with regularization

We generated a predictive model of SCI severity using the eigengenes (first principal components; Horvath and Dong, 2008) of the 16 SCI-specific gene modules as predictors. AIS at discharge from the hospital was used as the target outcome variable in a multinomial logistic regression model. To deal with the high number of predictors (16 modules), LASSO regularization was applied, using leave-one-out cross-validation to determine the regularization parameter (λ). The final model was chosen with λ producing a model with a misclassification error at 1 SD from the minimal misclassification error. The model was specified using the glmnet (Friedman et al., 2010) and the glmnetUtils (Microsoft and Ooi, 2020) packages in R. The model was assessed by confusion matrix metrics (Table S3 and Table S4) of internal prediction obtained using the caret R package (Kuhn, 2020). Overall accuracy (percentage of correct classification) was 72.7% with a 95% CI of 54.5–86.7%, resulting in significant accuracy (P value <0.0001) against random classification (no information rate of 36.4%). The uniform weighted overall accuracy (accounting for class unbalance) was 62.3%, with accuracy for AIS A = 83.3%, AIS B = 25%, AIS C = 50%, and AIS D = 90.9%. The same approach was performed for the model predicting the cervical vs. other NLI (Table S6 and Table S7). ROC curves for each AIS class were obtained by binarizing the problem (e.g., for AIS A, A = 1; B, C, D = 0) and rerunning the model as a binary classification. The curves were obtained using the roc() function of the pROC R package (Robin et al., 2011), and smoothing transformation was applied to each ROC curve using the smooth() function of the pROC R package.

### CIBERSORTx and module enrichment analysis

CIBERSORTx (Newman et al., 2019) is a machine learning algorithm that uses deconvolution methods to infer the proportions of cell types from gene expression patterns in bulk tissues. It is called digital cytometry because it performs an analogous function to regular flow cytometry without the need to physically isolate cells. We used the CIBERSORTx tool (https://cibersortx.stanford.edu/index.php) on all 58 of our samples and cross-referenced it with the LM22 signature (Newman et al., 2015) using 100 permutations. LM22 is a validated leukocyte gene signature matrix containing 547 genes that distinguish 22 human hematopoietic cell types. The output of the algorithm is the relative abundance of each of the 22 subtypes for all samples (Fig. 4, A and B). The cell types that have a P value <0.05 after Benjamini–Hochberg correction are shown in Fig. 4 C. For the calculation of leukocyte ratio estimates, we used the 22 subtypes from CIBERSORTx. While neutrophils and monocytes are among the 22 subtypes, lymphocytes had to be calculated. Lymphocyte counts were generated after summing the values of naive B cells, memory B cells, plasma cells, CD8 T cells, naive CD4 T cells, resting memory CD4 T cells, activated memory CD4 T cells, helper follicular T cells, regulatory T cells, γδ T cells, resting NK cells, and activated NK cells. One-way ANOVA was used to identify cell types that differ significantly among HCs, TCs, and SCIs. For enrichment analysis, modules were defined as all unique genes with positive *k*_ME_ values (Pearson correlation coefficients of module eigengenes; Horvath and Dong, 2008) that were significant after applying a Bonferroni correction for multiple comparisons (P < 0.05 / [no. genes × no. modules]). If a gene was significantly correlated with more than one module eigengene, it was assigned to the module for which it had the highest *k*_ME_ value. Enrichment analysis was performed for each gene set of interest with published human RNA-seq datasets (Newman et al., 2015; Watkins et al., 2009) using a one-sided Fisher's exact test as implemented by the fisher.test R function.

### Study approval

All the methods and protocols presented in this study were approved by the Institutional Review Board of the University of California, San Francisco (Institutional Review Board approval number: 15–16115). All participants of the study provided written informed consent before enrollment, and they are identified with a random identification specific for this manuscript only after removing any variable that could result in patient reidentification.

### Online supplemental material

Fig. S1 demonstrates the in-hospital flowchart of the SCI patients’ data collection. Fig. S2 shows the differential gene expression between HC, TC, and SCI in volcano plots and heatmap. Fig. S3 displays the GO enrichment analysis. Table S1 shows the basic demographic characteristics of the SCI patients and controls included in this study. Table S2 lists the 197 differentially expressed genes whose expression changes in an SCI severity-related manner. Table S3, Table S4, Table S6, and Table S7 summarize the statistical models used for AIS grade and NLI classification. Table S5 contains the neurological examination details of the 38 SCI patients used in this study. Table S8 shows the summary of the sequencing output of our 58 samples. All tables show summarized data. For detailed data for each individual subject, the data have been deposited to the Open Data Commons for Spinal Cord Injury (and can be accessed at https://doi.org/10.34945/F5QC7J).

## Supplementary Material

Table S1 shows demographic data for patients in the analysis.

Table S2 lists the 197 genes whose expression changes in an SCI severity–dependent manner.

Table S3 shows a confusion matrix.

Table S4 shows summary statistics of AIS grade predictive model.

Table S5 shows neurological examination of the SCI patients.

Table S6 shows the confusion matrix for the NLI predictive model.

Table S7 shows summary statistics of the NLI predictive model.

Table S8 shows sequenced samples output and quality metrics.

## References

[bib1] Adler, D., and D. Murdoch. 2019. rgl: 3D Visualization Using OpenGL. R package version 0.100.30*.* https://CRAN.R-project.org/package=rgl

[bib2] Andrews, S. 2010. FastQC: a quality control tool for high throughput sequence data. http://www.bioinformatics.babraham.ac.uk/projects/fastqc

[bib3] Biering-Sørensen, F., S. Alai, K. Anderson, S. Charlifue, Y. Chen, M. DeVivo, A.E. Flanders, L. Jones, N. Kleitman, A. Lans, . 2015. Common data elements for spinal cord injury clinical research: a National Institute for Neurological Disorders and Stroke project. Spinal Cord. 53:265–277. 10.1038/sc.2014.246 25665542 PMC4393777

[bib4] Blighe, K. 2019 a. EnhancedVolcano: Publication-ready volcano plots with enhanced colouring and labeling. R package version 1.2.0*.* https://github.com/kevinblighe/EnhancedVolcano

[bib5] Blighe, K. 2019 b. PCAtools: PCAtools: Everything Principal Components Analysis. R package version 1.1.10. https://github.com/kevinblighe/PCAtools

[bib6] Bloom, O., P.E. Herman, and A.M. Spungen. 2020. Systemic inflammation in traumatic spinal cord injury. Exp. Neurol. 325:113143. 10.1016/j.expneurol.2019.113143 31843491

[bib7] Choi, S.J., Y.H. Hong, S.M. Kim, J.Y. Shin, Y.J. Suh, and J.J. Sung. 2020. High neutrophil-to-lymphocyte ratio predicts short survival duration in amyotrophic lateral sclerosis. Sci. Rep. 10:428. 10.1038/s41598-019-57366-y 31949271 PMC6965090

[bib8] de Vries, A., and D.B. Ripley. 2016. ggdendro: Create Dendrograms and Tree Diagrams Using 'ggplot2'. R package version 0.1-20*.* https://CRAN.R-project.org/package=ggdendro

[bib9] Dhall, S.S., J. Haefeli, J.F. Talbott, A.R. Ferguson, W.J. Readdy, J.C. Bresnahan, M.S. Beattie, J.Z. Pan, G.T. Manley, and W.D. Whetstone. 2018. Motor Evoked Potentials Correlate With Magnetic Resonance Imaging and Early Recovery After Acute Spinal Cord Injury. Neurosurgery. 82:870–876. 10.1093/neuros/nyx320 28973360

[bib10] Eden, E., R. Navon, I. Steinfeld, D. Lipson, and Z. Yakhini. 2009. GOrilla: a tool for discovery and visualization of enriched GO terms in ranked gene lists. BMC Bioinformatics. 10:48. 10.1186/1471-2105-10-48 19192299 PMC2644678

[bib11] Ehsanian, R., J. Haefeli, N. Quach, J. Kosarchuk, D. Torres, E.D. Stuck, J. Endo, J.D. Crew, B. Dirlikov, J.C. Bresnahan, . 2020. Exploration of surgical blood pressure management and expected motor recovery in individuals with traumatic spinal cord injury. Spinal Cord. 58:377–386. 10.1038/s41393-019-0370-5 31649323 PMC7062632

[bib12] Elizei, S.S., and B.K. Kwon. 2017. Correction: The translational importance of establishing biomarkers of human spinal cord injury. Neural Regen. Res. 12:674. 10.4103/1673-5374.206661 28469645 PMC5399708

[bib13] Friedman, J., T. Hastie, and R. Tibshirani. 2010. Regularization Paths for Generalized Linear Models via Coordinate Descent. J. Stat. Softw. 33:1–22. 10.18637/jss.v033.i01 20808728 PMC2929880

[bib14] Guo, K., and B. McGregor. 2019. VennDetail: A package for visualization and extract details. R package version 1.0.1. https://github.com/guokai8/VennDetail

[bib15] Harris, P.A., R. Taylor, R. Thielke, J. Payne, N. Gonzalez, and J.G. Conde. 2009. Research electronic data capture (REDCap)--a metadata-driven methodology and workflow process for providing translational research informatics support. J. Biomed. Inform. 42:377–381. 10.1016/j.jbi.2008.08.010 18929686 PMC2700030

[bib16] Herman, P., A. Stein, K. Gibbs, I. Korsunsky, P. Gregersen, and O. Bloom. 2018. Persons with Chronic Spinal Cord Injury Have Decreased Natural Killer Cell and Increased Toll-Like Receptor/Inflammatory Gene Expression. J. Neurotrauma. 35:1819–1829. 10.1089/neu.2017.5519 29310515 PMC6033303

[bib17] Hong, J., A. Chang, M.M. Zavvarian, J. Wang, Y. Liu, and M.G. Fehlings. 2018. Level-Specific Differences in Systemic Expression of Pro- and Anti-Inflammatory Cytokines and Chemokines after Spinal Cord Injury. Int. J. Mol. Sci. 19:2167. 10.3390/ijms19082167 PMC6122077 30044384

[bib18] Horvath, S., and J. Dong. 2008. Geometric interpretation of gene coexpression network analysis. PLOS Comput. Biol. 4:e1000117. 10.1371/journal.pcbi.1000117 18704157 PMC2446438

[bib19] Hulme, C.H., S.J. Brown, H.R. Fuller, J. Riddell, A. Osman, J. Chowdhury, N. Kumar, W.E. Johnson, and K.T. Wright. 2017. The developing landscape of diagnostic and prognostic biomarkers for spinal cord injury in cerebrospinal fluid and blood. Spinal Cord. 55:114–125. 10.1038/sc.2016.174 27995945

[bib20] Johnson, W.E., C. Li, and A. Rabinovic. 2007. Adjusting batch effects in microarray expression data using empirical Bayes methods. Biostatistics. 8:118–127. 10.1093/biostatistics/kxj037 16632515

[bib21] Kelley, K.W., H. Nakao-Inoue, A.V. Molofsky, and M.C. Oldham. 2018. Variation among intact tissue samples reveals the core transcriptional features of human CNS cell classes. Nat. Neurosci. 21:1171–1184. 10.1038/s41593-018-0216-z 30154505 PMC6192711

[bib22] Kim, D., G. Pertea, C. Trapnell, H. Pimentel, R. Kelley, and S.L. Salzberg. 2013. TopHat2: accurate alignment of transcriptomes in the presence of insertions, deletions and gene fusions. Genome Biol. 14:R36. 10.1186/gb-2013-14-4-r36 23618408 PMC4053844

[bib23] Krijthe, J.H. 2015. Rtsne: T-Distributed Stochastic Neighbor Embedding using a Barnes-Hut Implementation. https://github.com/jkrijthe/Rtsne

[bib24] Kuhn, M. 2020. caret: Classification and Regression Training. R package version 6.0-85. https://CRAN.R-project.org/package=caret

[bib25] Kwon, B.K., F. Streijger, N. Fallah, V.K. Noonan, L.M. Bélanger, L. Ritchie, S.J. Paquette, T. Ailon, M.C. Boyd, J. Street, . 2017. Cerebrospinal Fluid Biomarkers To Stratify Injury Severity and Predict Outcome in Human Traumatic Spinal Cord Injury. J. Neurotrauma. 34:567–580. 10.1089/neu.2016.4435 27349274

[bib26] Kwon, B.K., O. Bloom, I.B. Wanner, A. Curt, J.M. Schwab, J. Fawcett, and K.K. Wang. 2019. Neurochemical biomarkers in spinal cord injury. Spinal Cord. 57:819–831. 10.1038/s41393-019-0319-8 31273298

[bib27] Langfelder, P., and S. Horvath. 2007. Eigengene networks for studying the relationships between co-expression modules. BMC Syst. Biol. 1:54. 10.1186/1752-0509-1-54 18031580 PMC2267703

[bib28] Leek, J.T., W.E. Johnson, H.S. Parker, A.E. Jaffe, and J.D. Storey. 2012. The sva package for removing batch effects and other unwanted variation in high-throughput experiments. Bioinformatics. 28:882–883. 10.1093/bioinformatics/bts034 22257669 PMC3307112

[bib29] Leister, I., T. Haider, G. Mattiassich, J.L.K. Kramer, L.D. Linde, A. Pajalic, L. Grassner, B. Altendorfer, H. Resch, S. Aschauer-Wallner, and L. Aigner. 2020. Biomarkers in Traumatic Spinal Cord Injury-Technical and Clinical Considerations: A Systematic Review. Neurorehabil. Neural Repair. 34:95–110. 10.1177/1545968319899920 31971869

[bib30] Liao, Y., G.K. Smyth, and W. Shi. 2014. featureCounts: an efficient general purpose program for assigning sequence reads to genomic features. Bioinformatics. 30:923–930. 10.1093/bioinformatics/btt656 24227677

[bib31] Light, M., K.H. Minor, P. DeWitt, K.H. Jasper, and S.J. Davies. 2012. Multiplex array proteomics detects increased MMP-8 in CSF after spinal cord injury. J. Neuroinflammation. 9:122. 10.1186/1742-2094-9-122 22687332 PMC3439361

[bib32] Lim, S.B., W. Di Lee, J. Vasudevan, W.T. Lim, and C.T. Lim. 2019. Liquid biopsy: one cell at a time. NPJ Precis. Oncol. 3:23. 10.1038/s41698-019-0095-0 31602399 PMC6775080

[bib33] Liu, Y., J. Zhou, and K.P. White. 2014. RNA-seq differential expression studies: more sequence or more replication? Bioinformatics. 30:301–304. 10.1093/bioinformatics/btt688 24319002 PMC3904521

[bib34] Lubieniecka, J.M., F. Streijger, J.H. Lee, N. Stoynov, J. Liu, R. Mottus, T. Pfeifer, B.K. Kwon, J.R. Coorssen, L.J. Foster, . 2011. Biomarkers for severity of spinal cord injury in the cerebrospinal fluid of rats. PLoS One. 6:e19247. 10.1371/journal.pone.0019247 21559420 PMC3084780

[bib35] Marbourg, J.M., A. Bratasz, X. Mo, and P.G. Popovich. 2017. Spinal Cord Injury Suppresses Cutaneous Inflammation: Implications for Peripheral Wound Healing. J. Neurotrauma. 34:1149–1155. 10.1089/neu.2016.4611 27650169 PMC5359642

[bib36] Maynard, F.M. Jr., M.B. Bracken, G. Creasey, J.F. Ditunno Jr., W.H. Donovan, T.B. Ducker, S.L. Garber, R.J. Marino, S.L. Stover, C.H. Tator, . American Spinal Injury Association. 1997. International Standards for Neurological and Functional Classification of Spinal Cord Injury. Spinal Cord. 35:266–274. 10.1038/sj.sc.3100432 9160449

[bib37] McCoy, D.B., S.M. Dupont, C. Gros, J. Cohen-Adad, R.J. Huie, A. Ferguson, X. Duong-Fernandez, L.H. Thomas, V. Singh, J. Narvid, . TRACK-SCI Investigators. 2019. Convolutional Neural Network-Based Automated Segmentation of the Spinal Cord and Contusion Injury: Deep Learning Biomarker Correlates of Motor Impairment in Acute Spinal Cord Injury. AJNR Am. J. Neuroradiol. 40:737–744. 10.3174/ajnr.A6020 30923086 PMC7048524

[bib38] Microsoft, and H. Ooi. 2020. glmnetUtils: Utilities for 'Glmnet'. R package version 1.1.5. https://CRAN.R-project.org/package=glmnetUtils

[bib39] *Nature Cancer*. 2020. Spotlight on cancer genomics. *Nature Cancer*. 1:265–266. 10.1038/s43018-020-0052-4 35122034

[bib40] Newman, A.M., C.L. Liu, M.R. Green, A.J. Gentles, W. Feng, Y. Xu, C.D. Hoang, M. Diehn, and A.A. Alizadeh. 2015. Robust enumeration of cell subsets from tissue expression profiles. Nat. Methods. 12:453–457. 10.1038/nmeth.3337 25822800 PMC4739640

[bib41] Newman, A.M., C.B. Steen, C.L. Liu, A.J. Gentles, A.A. Chaudhuri, F. Scherer, M.S. Khodadoust, M.S. Esfahani, B.A. Luca, D. Steiner, . 2019. Determining cell type abundance and expression from bulk tissues with digital cytometry. Nat. Biotechnol. 37:773–782. 10.1038/s41587-019-0114-2 31061481 PMC6610714

[bib42] Norris-Baker, C., M.A. Stephens, D.H. Rintala, and E.P. Willems. 1981. Patient behavior as a predictor of outcomes in spinal cord injury. Arch. Phys. Med. Rehabil. 62:602–608. 7316720

[bib43] Oldham, M.C., S. Horvath, and D.H. Geschwind. 2006. Conservation and evolution of gene coexpression networks in human and chimpanzee brains. Proc. Natl. Acad. Sci. USA. 103:17973–17978. 10.1073/pnas.0605938103 17101986 PMC1693857

[bib44] Oldham, M.C., G. Konopka, K. Iwamoto, P. Langfelder, T. Kato, S. Horvath, and D.H. Geschwind. 2008. Functional organization of the transcriptome in human brain. Nat. Neurosci. 11:1271–1282. 10.1038/nn.2207 18849986 PMC2756411

[bib45] Oldham, M.C., P. Langfelder, and S. Horvath. 2012. Network methods for describing sample relationships in genomic datasets: application to Huntington’s disease. BMC Syst. Biol. 6:63. 10.1186/1752-0509-6-63 22691535 PMC3441531

[bib46] Ooms, J. 2020. magick: Advanced Graphics and Image-Processing in R. R package version 2.3. https://CRAN.R-project.org/package=magick

[bib47] Prüss, H., A. Tedeschi, A. Thiriot, L. Lynch, S.M. Loughhead, S. Stutte, I.B. Mazo, M.A. Kopp, B. Brommer, C. Blex, . 2017. Spinal cord injury-induced immunodeficiency is mediated by a sympathetic-neuroendocrine adrenal reflex. Nat. Neurosci. 20:1549–1559. 10.1038/nn.4643 28920935

[bib48] Quan, X.Q., R.C. Wang, Q. Zhang, C.T. Zhang, and L. Sun. 2020. The predictive value of lymphocyte-to-monocyte ratio in the prognosis of acute coronary syndrome patients: a systematic review and meta-analysis. BMC Cardiovasc. Disord. 20:338. 10.1186/s12872-020-01614-x 32669086 PMC7362430

[bib49] R Core Team. 2019. R: A language and environment for statistical computing. R Foundation for Statistical Computing, Vienna, Austria. http://www.R-project.org/. R version 4.0.3 installed on October 10, 2020.

[bib50] R Studio Team. 2015. RStudio: Integrated Development for R. RStudio, Inc., Boston, MA. http://www.rstudio.com/. R Studio version 1.2.5042 installed on May 18, 2020.

[bib51] Raman, K., M.J. O’Donnell, A. Czlonkowska, Y.C. Duarte, P. Lopez-Jaramillo, E. Peñaherrera, M. Sharma, A. Shoamanesh, M. Skowronska, S. Yusuf, and G. Paré. 2016. Peripheral Blood MCEMP1 Gene Expression as a Biomarker for Stroke Prognosis. Stroke. 47:652–658. 10.1161/STROKEAHA.115.011854 26846866

[bib52] Rich, B. 2018. table1: Tables of Descriptive Statistics in HTML. R package version 1.1. https://CRAN.R-project.org/package=table1

[bib53] Ritchie, M.E., B. Phipson, D. Wu, Y. Hu, C.W. Law, W. Shi, and G.K. Smyth. 2015. limma powers differential expression analyses for RNA-sequencing and microarray studies. Nucleic Acids Res. 43:e47. 10.1093/nar/gkv007 25605792 PMC4402510

[bib54] Roberts, T.T., G.R. Leonard, and D.J. Cepela. 2017. Classifications In Brief: American Spinal Injury Association (ASIA) Impairment Scale. Clin. Orthop. Relat. Res. 475:1499–1504. 10.1007/s11999-016-5133-4 27815685 PMC5384910

[bib55] Robin, X., N. Turck, A. Hainard, N. Tiberti, F. Lisacek, J.C. Sanchez, and M. Müller. 2011. pROC: an open-source package for R and S+ to analyze and compare ROC curves. BMC Bioinformatics. 12:77. 10.1186/1471-2105-12-77 21414208 PMC3068975

[bib56] Robinson, M.D., D.J. McCarthy, and G.K. Smyth. 2010. edgeR: a Bioconductor package for differential expression analysis of digital gene expression data. Bioinformatics. 26:139–140. 10.1093/bioinformatics/btp616 19910308 PMC2796818

[bib57] Sengupta, M.B., M. Basu, S. Iswarari, K.K. Mukhopadhyay, K.P. Sardar, B. Acharyya, P.K. Mohanty, and D. Mukhopadhyay. 2014. CSF proteomics of secondary phase spinal cord injury in human subjects: perturbed molecular pathways post injury. PLoS One. 9:e110885. 10.1371/journal.pone.0110885 25350754 PMC4211693

[bib58] Supek, F., M. Bošnjak, N. Škunca, and T. Šmuc. 2011. REVIGO summarizes and visualizes long lists of gene ontology terms. PLoS One. 6:e21800. 10.1371/journal.pone.0021800 21789182 PMC3138752

[bib59] Talbott, J.F., W.D. Whetstone, W.J. Readdy, A.R. Ferguson, J.C. Bresnahan, R. Saigal, G.W. Hawryluk, M.S. Beattie, M.C. Mabray, J.Z. Pan, . 2015. The Brain and Spinal Injury Center score: a novel, simple, and reproducible method for assessing the severity of acute cervical spinal cord injury with axial T2-weighted MRI findings. J. Neurosurg. Spine. 23:495–504. 10.3171/2015.1.SPINE141033 26161519

[bib60] Targonski, C.A., C.A. Shearer, B.T. Shealy, M.C. Smith, and F.A. Feltus. 2019. Uncovering biomarker genes with enriched classification potential from Hallmark gene sets. Sci. Rep. 9:9747. 10.1038/s41598-019-46059-1 31278367 PMC6611793

[bib61] Tigchelaar, S., F. Streijger, S. Sinha, S. Flibotte, N. Manouchehri, K. So, K. Shortt, E. Okon, M.A. Rizzuto, I. Malenica, . 2017. Serum MicroRNAs Reflect Injury Severity in a Large Animal Model of Thoracic Spinal Cord Injury. Sci. Rep. 7:1376. 10.1038/s41598-017-01299-x 28469141 PMC5431108

[bib62] Tigchelaar, S., R. Gupta, C.P. Shannon, F. Streijger, S. Sinha, S. Flibotte, M.A. Rizzuto, J. Street, S. Paquette, T. Ailon, . 2019. MicroRNA Biomarkers in Cerebrospinal Fluid and Serum Reflect Injury Severity in Human Acute Traumatic Spinal Cord Injury. J. Neurotrauma. 36:2358–2371. 10.1089/neu.2018.6256 30827169

[bib63] Tsolinas, R.E., J.F. Burke, A.M. DiGiorgio, L.H. Thomas, X. Duong-Fernandez, M.H. Harris, J.K. Yue, E.A. Winkler, C.G. Suen, L.U. Pascual, . 2020. Transforming Research and Clinical Knowledge in Spinal Cord Injury (TRACK-SCI): an overview of initial enrollment and demographics. Neurosurg. Focus. 48:E6. 10.3171/2020.2.FOCUS191030 32357323

[bib64] Watkins, N.A., A. Gusnanto, B. de Bono, S. De, D. Miranda-Saavedra, D.L. Hardie, W.G. Angenent, A.P. Attwood, P.D. Ellis, W. Erber, . Bloodomics Consortium. 2009. A HaemAtlas: characterizing gene expression in differentiated human blood cells. Blood. 113:e1–e9. 10.1182/blood-2008-06-162958 19228925 PMC2680378

[bib65] Wickham, H. 2016. ggplot2: Elegant Graphics for Data Analysis. Springer-Verlag*, *New York.

[bib66] Wickham, H., R. François, L. Henry, and K. Müller. 2020. dplyr: A Grammar of Data Manipulation. R package version 0.8.4. https://CRAN.R-project.org/package=dplyr

[bib67] Wilke, C.O. 2019. cowplot: Streamlined Plot Theme and Plot Annotations for 'ggplot2'. R package version 1.0.0. https://CRAN.R-project.org/package=cowplot

[bib68] Zhao, J.L., S.T. Lai, Z.Y. Du, J. Xu, Y.R. Sun, Q. Yuan, X. Wu, Z.Q. Li, J. Hu, and R. Xie. 2020. Circulating neutrophil-to-lymphocyte ratio at admission predicts the long-term outcome in acute traumatic cervical spinal cord injury patients. BMC Musculoskelet. Disord. 21:548. 10.1186/s12891-020-03556-z 32799840 PMC7429795

