# Supplementary material for: Diagnostic blood RNA profiles for human acute spinal cord injury
Source: J Exp Med. 2021 Jan 29;218(3):e20201795. doi: 10.1084/jem.20201795 (PMC7852457; doi:10.1084/jem.20201795)
Supplement: Table S1 — shows demographic data for patients in the analysis. [file JEM_20201795_TableS1.docx]

Table S1. **Demographic data for patients in the analysis**

|  | HC (*n* = 10) | TC (*n* = 10) | SCI (*n* = 38) |
| --- | --- | --- | --- |
| Sex |  |  |  |
| Male | 8 (80.0%) | 6 (60.0%) | 25 (65.8%) |
| Female | 2 (20.0%) | 4 (40.0%) | 13 (34.2%) |
| **Race** |  |  |  |
| Asian | 4 (40.0%) | 2 (20.0%) | 10 (26.3%) |
| Black or African-American | 0 (0%) | 0 (0%) | 5 (13.2%) |
| Hispanic | 0 (0%) | 1 (10.0%) | 3 (7.9%) |
| White | 4 (40.0%) | 4 (40.0%) | 17 (44.7%) |
| Other | 0 (0%) | 3 (30.0%) | 0 (0%) |
| Unknown | 2 (20.0%) | 0 (0%) | 3 (7.9%) |
| **Age (years)** |  |  |  |
| Mean ± SD | 49.4 ± 12.2 | 41.7 ± 18.0 | 55.3 ± 20.0 |
| Median [Min, Max] | 51.0 (31.0, 67.0) | 42.0 (22.0, 78.0) | 53.5 (20.0, 89.0) |
| Missing | 1 (10.0%) | 0 (0%) | 0 (0%) |
| **Injury Severity Score** |  |  |  |
| Mean ± SD | NA ± NA | 6.57 ± 4.35 | 27.8 ± 13.2 |
| Median [Min, Max] | NA (NA, NA) | 5.00 (1.00, 14.0) | 26.5 (9.00, 75.0) |
| Missing | 10 (100%) | 3 (30.0%) | 2 (5.3%) |
| **Prior CNS pathology** |  |  |  |
| Yes | 0 (0%) | 0 (0%) | 12 (31.6%) |
| No | 10 (100%) | 10 (100%) | 21 (55.3%) |
| Unknown | 0 (0%) | 0 (0%) | 5 (13.2%) |
| **Concurrent TBI** |  |  |  |
| Yes | 0 (0%) | 0 (0%) | 8 (21.1%) |
| No | 10 (100%) | 10 (100%) | 28 (73.7%) |
| Unknown | 0 (0%) | 0 (0%) | 2 (5.3%) |
| **Time from blood draw (hours after injury)** |  |  |  |
| Mean ± SD | NA ± NA | 21.5 ± 5.75 | 30.3 ± 18.9 |
| Median [Min, Max] | NA (NA, NA) | 20.0 (15.0, 32.0) | 23.0 (5.00, 97.0) |
| Missing | 10 (100%) | 4 (40.0%) | 0 (0%) |
