# Supplementary material for: Diagnostic blood RNA profiles for human acute spinal cord injury
Source: J Exp Med. 2021 Jan 29;218(3):e20201795. doi: 10.1084/jem.20201795 (PMC7852457; doi:10.1084/jem.20201795)
Supplement: Table S2 — lists the 197 genes whose expression changes in an SCI severity–dependent manner. [file JEM_20201795_TableS2.docx]

Table S2. **List of the 197 genes whose expression changes in an SCI severity–dependent manner**

| Gene | Name |
| --- | --- |
|  |  |
| **SCI severity  gene ** | |
| *ADAMTS3* | *ADAM metallopeptidase with thrombospondin type 1 motif 3* |
| *ALDH1A2* | *Aldehyde dehydrogenase 1 family member A2* |
| *ANKRD34B* | *Ankyrin repeat domain 34B* |
| *ANO10* | *Anoctamin 10* |
| *AREG* | *Amphiregulin* |
| *ARG1* | *Arginase 1* |
| *ARMC12* | *Armadillo repeat–containing 12* |
| *ASPH* | *Aspartate β-hydroxylase* |
| *BCL2A1* | *BCL2-related protein A1* |
| *C3AR1* | *Complement C3a receptor 1* |
| *C8orf88* | *Chromosome 8 open reading frame 88* |
| *CAMP* | *Cathelicidin antimicrobial peptide* |
| *CARD6* | *Caspase recruitment domain family member 6* |
| *CCDC183-AS1* | *CCDC183 antisense RNA 1* |
| *CCM2L* | *CCM2-like scaffold protein* |
| *CDKL5* | *Cyclin-dependent kinase–like 5* |
| *CHPT1* | *Choline phosphotransferase 1* |
| *CLEC6A* | *C-type lectin domain–containing 6A* |
| *CRISP2* | *Cysteine-rich secretory protein 2* |
| *CRISP3* | *Cysteine-rich secretory protein 3* |
| *DACH1* | *Dachshund family transcription factor 1* |
| *DHRS9* | *Dehydrogenase/reductase 9* |
| *DRAM1* | *DNA damage–regulated autophagy modulator 1* |
| *DUSP13* | *Dual-specificity phosphatase 13* |
| *EFNA1* | *Ephrin A1* |
| *FCER1G* | *Fc fragment of IgE receptor Ig* |
| *FKBP5* | *FKBP prolyl isomerase 5* |
| *FMN1* | *Formin 1* |
| *FOLR3* | *Folate receptor γ* |
| *FRMD4B* | *FERM domain–containing 4B* |
| *GABRR2* | *γ-Aminobutyric acid type A receptor subunit ρ2* |
| *GADD45A* | *Growth arrest and DNA damage–inducible α* |
| *GPR160* | *G protein–coupled receptor 160* |
| *GYG1* | *Glycogenin 1* |
| *HMGB2* | *High-mobility group box 2* |
| *HN1* | *Jupiter microtubule–associated homolog 1* |
| *HP* | *Haptoglobin* |
| *HPGD* | *15-Hydroxyprostaglandin dehydrogenase* |
| *HTATIP2* | *HIV-1 Tat interactive protein 2* |
| *IDI2-AS1* | *IDI2 antisense RNA 1* |
| *IFITM3* | *Interferon-induced transmembrane protein 3* |
| *IL18RAP* | *Interleukin 18 receptor accessory protein* |
| *IL1R2* | *Interleukin 1 receptor type 2* |
| *ITGA9* | *Integrin subunit α 9* |
| *KBTBD7* | *Kelch repeat and BTB domain–containing 7* |
| *KCNE1* | *Potassium voltage-gated channel subfamily E regulatory subunit 1* |
| *KCNG2* | *Potassium voltage-gated channel modifier subfamily G member 2* |
| *KL* | *Klotho* |
| *LCN2* | *Lipocalin 2* |
| *LDHA* | *Lactate dehydrogenase A* |
| *LHFP* | *LHFPL tetraspan subfamily member 6* |
| *LILRB4* | *Leukocyte immunoglobulin–like receptor B4* |
| *LIN7A* | *Lin-7 homolog A, crumbs cell polarity complex component* |
| *LINC00671* | *Long intergenic non-protein coding RNA 671* |
| *LINC01094* | *Long intergenic non-protein coding RNA 1094* |
| *LINC01271* | *Long intergenic non-protein coding RNA 1271* |
| *LINC01581* | *Long intergenic non-protein coding RNA 1581* |
| *LOC100289650* |  |
| *LOC101927153* |  |
| *LOC102546294* |  |
| *LOC642943* |  |
| *LRRC70* | *Leucine-rich repeat–containing 70* |
| *LTB4R* | *Leukotriene B4 receptor* |
| *MAOA* | *Monoamine oxidase A* |
| *MAP2K6* | *Mitogen-activated protein kinase kinase 6* |
| *MB21D1* | *Cyclic GMP-AMP synthase* |
| *MCEMP1* | *Mast cell–expressed membrane protein 1* |
| *METTL9* | *Methyltransferase like 9* |
| *MILR1* | *Mast cell immunoglobulin–like receptor 1* |
| *MIR1273D* | *MicroRNA 1273d* |
| *MIR618* | *MicroRNA 618* |
| *MIR6818* | *MicroRNA 6818* |
| *MMP8* | *Matrix metallopeptidase 8* |
| *MS4A4A* | *Membrane spanning 4-domains A4A* |
| *MSRB2* | *Methionine sulfoxide reductase B2* |
| *NANS* | *N-acetylneuraminate synthase* |
| *NEDD4* | *NEDD4 E3 ubiquitin protein ligase* |
| *OLFM4* | *Olfactomedin 4* |
| *OLR1* | *Oxidized low-density lipoprotein receptor 1* |
| *OR52W1* | *Olfactory receptor family 52 subfamily W member 1* |
| *OSBPL1A* | *Oxysterol-binding protein–like 1A* |
| *PCOLCE2* | *Procollagen C-endopeptidase enhancer 2* |
| *PDSS1* | *Decaprenyl diphosphate synthase subunit 1* |
| *PLB1* | *Phospholipase B1* |
| *PLP2* | *Proteolipid protein 2* |
| *PPARG* | *Peroxisome proliferator–activated receptor γ* |
| *PPP1R1A* | *Protein phosphatase 1 regulatory inhibitor subunit 1A* |
| *PRG1* | *Immediate early response 3* |
| *PRTN3* | *Proteinase 3* |
| *PTX3* | *Pentraxin 3* |
| *RABGEF1* | *RAB guanine nucleotide exchange factor 1* |
| *RNASE1* | *Ribonuclease A family member 1, pancreatic* |
| *RNASE4* | *Ribonuclease A family member 4* |
| *RNF217* | *Ring-finger protein 217* |
| *S100P* | *S100 calcium–binding protein P* |
| *SAMSN1* | *SAM domain, SH3 domain, and nuclear localization signals 1* |
| *SAP30* | *Sin3A-associated protein 30* |
| *SCN9A* | *Sodium voltage-gated channel α subunit 9* |
| *SCPEP1* | *Serine carboxypeptidase 1* |
| *SDHAF3* | *Succinate dehydrogenase–complex assembly factor 3* |
| *SLC25A24* | *Solute carrier family 25 member 24* |
| *SLC38A11* | *Solute carrier family 38 member 11* |
| *SLCO4A1* | *Solute carrier organic anion transporter family member 4A1* |
| *SNX3* | *Sorting nexin 3* |
| *STBD1* | *Starch-binding domain 1* |
| *SYCP2* | *Synaptonemal complex protein 2* |
| *TCN1* | *Transcobalamin 1* |
| *TIMP1* | *TIMP metallopeptidase inhibitor 1* |
| *TMCO3* | *Transmembrane and coiled-coil domains 3* |
| *TMIGD3* | *Transmembrane and immunoglobulin–domain containing 3* |
| *TPST1* | *Tyrosylprotein sulfotransferase 1* |
| *TREML3P* | *Triggering receptor expressed on myeloid cells–like 3, pseudogene* |
| *TTC8* | *Tetratricopeptide repeat domain 8* |
| *TTN-AS1* | *TTN antisense RNA 1* |
| *UBE2J1* | *Ubiquitin conjugating enzyme E2 J1* |
| *UGCG* | *UDP-glucose ceramide glucosyltransferase* |
| *UPB1* | *β-Ureidopropionase 1* |
| **SCI severity  gene ** | |
| *A2MP1* | *α-2-Macroglobulin pseudogene 1* |
| *AFAP1* | *Actin filament–associated protein 1* |
| *AHNAK2* | *AHNAK nucleoprotein 2* |
| *AKAP11* | *A-kinase anchoring protein 11* |
| *ALOX12P2* | *Arachidonate 12-lipoxygenase pseudogene 2* |
| *ARL10* | *ADP ribosylation factor–like GTPase 10* |
| *ASB2* | *Ankyrin repeat and SOCS box–containing 2* |
| *ATG9B* | *Autophagy-related 9B* |
| *ATP10A* | *ATPase phospholipid–transporting 10A (putative)* |
| *ATP6V0E2* | *ATPase H^+^–transporting V0 subunit e2* |
| *AXIN2* | *Axin 2* |
| *BCL9L* | *BCL9-like* |
| *BTBD11* | *BTB domain–containing 11* |
| *CACNA1C-AS1* | *CACNA1C antisense RNA 1* |
| *CACNA1I* | *Calcium voltage-gated channel subunit α1 I* |
| *CCDC88C* | *Coiled-coil domain–containing 88C* |
| *CD6* | *CD6 molecule* |
| *CD74* | *CD74 molecule* |
| *CELSR1* | *Cadherin EGF LAG seven-pass G-type receptor 1* |
| *COLGALT2* | *Collagen β(1-O)galactosyltransferase 2* |
| *CRLF1* | *Cytokine receptor–like factor 1* |
| *CTSF* | *Cathepsin F* |
| *CTSW* | *Cathepsin W* |
| *CXCL10* | *C-X-C motif chemokine ligand 10* |
| *DCANP1* | *Dendritic cell–associated nuclear protein* |
| *DEPDC7* | *DEP domain–containing 7* |
| *DFNB59* | *Pejvakin* |
| *EEF1A1* | *Eukaryotic translation elongation factor 1 α 1* |
| *ESYT1* | *Extended synaptotagmin 1* |
| *EVL* | *Enah/Vasp-like* |
| *FAM102A* | *Family with sequence similarity 102 member A* |
| *FAM132B* | *Erythroferrone* |
| *FAM150B* | *ALK and LTK ligand 2* |
| *FBXL16* | *F-box and leucine-rich repeat protein 16* |
| *FCRL6* | *Fc receptor–like 6* |
| *GFRA2* | *GDNF family receptor α 2* |
| *GLB1L2* | *Galactosidase β 1–like 2* |
| *GZMH* | *Granzyme H* |
| *HLA-DPB1* | *Major histocompatibility complex, class II, DP β 1* |
| *HSPG2* | *Heparan sulfate proteoglycan 2* |
| *IGSF9B* | *Immunoglobulin superfamily member 9B* |
| *KCNA6* | *Potassium voltage-gated channel subfamily A member 6* |
| *KDF1* | *Keratinocyte differentiation factor 1* |
| *KLHDC7A* | *Kelch domain–containing 7A* |
| *KLRD1* | *Killer cell lectin–like receptor D1* |
| *KNG1* | *Kininogen 1* |
| *LANCL1-AS1* | *LANCL1 antisense RNA 1* |
| *LGR6* | *Leucine-rich repeat–containing G protein-coupled receptor 6* |
| *LINC01278* | *Long intergenic non-protein coding RNA 1278* |
| *LOC100507091* |  |
| *LRRN3* | *Leucine-rich repeat neuronal 3* |
| *MLLT6* | *MLLT6, PHD finger–containing* |
| *MYBPH* | *Myosin-binding protein H* |
| *MYCBPAP* | *MYCBP-associated protein* |
| *MYO6* | *Myosin VI* |
| *NUP210* | *Nucleoporin 210* |
| *OLFM1* | *Olfactomedin 1* |
| *PASK* | *PAS domain–containing serine/threonine kinase* |
| *PDGFRB* | *Platelet-derived growth factor receptor β* |
| *PKIA-AS1* | *PKIA antisense RNA 1* |
| *PPP1R16B* | *Protein phosphatase 1 regulatory subunit 16B* |
| *PRSS33* | *Serine protease 33* |
| *PTGS2* | *Prostaglandin-endoperoxide synthase 2* |
| *RFX7* | *Regulatory factor X7* |
| *RNF125* | *Ring-finger protein 125* |
| *RPGRIP1L* | *RPGRIP1-like* |
| *S1PR5* | *Sphingosine-1-phosphate receptor 5* |
| *SARDH* | *Sarcosine dehydrogenase* |
| *SCART1* | *Scavenger receptor family member expressed on T cells 1* |
| *SERPINA2* | *Serpin family A member 2 (gene/pseudogene)* |
| *SLC25A23* | *Solute carrier family 25 member 23* |
| *SPATA13-AS1* | *SPATA13 antisense RNA 1* |
| *SPN* | *Sialophorin* |
| *SPOCK2* | *SPARC- (osteonectin-), cwcv-, and kazal-like domains proteoglycan 2* |
| *TMEM176A* | *Transmembrane protein 176A* |
| *TMIE* | *Transmembrane inner ear* |
| *TTC3P1* | *Tetratricopeptide repeat domain 3 pseudogene 1* |
| *UBASH3A* | *Ubiquitin-associated and SH3 domain–containing A* |
| *UTRN* | *Utrophin* |
| *ZNF703* | *Zinc finger protein 703* |
