# Supplementary material for: Diagnostic blood RNA profiles for human acute spinal cord injury
Source: J Exp Med. 2021 Jan 29;218(3):e20201795. doi: 10.1084/jem.20201795 (PMC7852457; doi:10.1084/jem.20201795)
Supplement: Table S3 — shows a confusion matrix. [file JEM_20201795_TableS3.docx]

Table S3. **Confusion matrix**

|  | Reference | | | | |
| --- | --- | --- | --- | --- | --- |
| Prediction |  | A | B | C | D |
|  | **A** | 10 | 2 | 2 | 1 |
|  | **B** | 0 | 1 | 0 | 0 |
|  | **C** | 0 | 0 | 3 | 0 |
|  | **D** | 2 | 1 | 1 | 10 |

This table, known as a confusion matrix, shows the performance of our model. One can see that from the 12 AIS A patients, our model predicts 10 of them correctly (sensitivity = 83.3%) but also misclassifies five more patients as A (specificity = 76.2%). For AIS B, C, and D, the sensitivity is 25%, 50%, and 90.9%, respectively, and the specificity is 100%, 100%, and 81.2% respectively. Overall, the accuracy of our model is 72.7% (P = 2.35 × 10^−5^).
