# Supplementary material for: Diagnostic blood RNA profiles for human acute spinal cord injury
Source: J Exp Med. 2021 Jan 29;218(3):e20201795. doi: 10.1084/jem.20201795 (PMC7852457; doi:10.1084/jem.20201795)
Supplement: Table S4 — shows summary statistics of AIS grade predictive model. [file JEM_20201795_TableS4.docx]

Table S4. **Summary statistics of AIS grade predictive model**

|  | A | B | C | D |
| --- | --- | --- | --- | --- |
| Sensitivity | 0.833 | 0.250 | 0.500 | 0.909 |
| Specificity | 0.762 | 1.000 | 1.000 | 0.818 |
| Positive predictive value | 0.667 | 1.000 | 1.000 | 0.714 |
| Negative predictive value | 0.889 | 0.906 | 0.900 | 0.947 |
| Precision | 0.667 | 1.000 | 1.000 | 0.714 |
| Recall | 0.833 | 0.250 | 0.500 | 0.909 |
| F1 | 0.741 | 0.400 | 0.667 | 0.800 |
| Prevalence | 0.364 | 0.121 | 0.182 | 0.333 |
| Detection rate | 0.303 | 0.030 | 0.091 | 0.303 |
| Detection prevalence | 0.455 | 0.030 | 0.091 | 0.424 |
| Balanced accuracy | 0.798 | 0.625 | 0.750 | 0.864 |
| Model accuracy \| weighted accuracy |  | 0.727 \| 0.623 | | |
