# Supplementary material for: Diagnostic blood RNA profiles for human acute spinal cord injury
Source: J Exp Med. 2021 Jan 29;218(3):e20201795. doi: 10.1084/jem.20201795 (PMC7852457; doi:10.1084/jem.20201795)
Supplement: Table S5 — shows neurological examination of the SCI patients. [file JEM_20201795_TableS5.docx]

Table S5. **Neurological examination of the SCI patients**

|  | A (*n* = 12) | B (*n* = 4) | C (*n* = 6) | D (*n* = 11) | Overall (*n* = 33) |
| --- | --- | --- | --- | --- | --- |
| Level of injury |  |  |  |  |  |
| Cervical | 2 (16.7%) | 3 (75.0%) | 3 (50.0%) | 10 (90.9%) | 18 (54.5%) |
| Thoracic | 8 (66.7%) | 1 (25.0%) | 1 (16.7%) | 0 (0%) | 10 (30.3%) |
| Lumbar | 1 (8.3%) | 0 (0%) | 0 (0%) | 1 (9.1%) | 2 (6.1%) |
| Unable to determine | 1 (8.3%) | 0 (0%) | 2 (33.3%) | 0 (0%) | 3 (9.1%) |
| **AIS actual or estimate** |  |  |  |  |  |
| Actual | 7 (58.3%) | 1 (25.0%) | 3 (50.0%) | 9 (81.8%) | 20 (60.6%) |
| Estimate | 5 (41.7%) | 3 (75.0%) | 3 (50.0%) | 2 (18.2%) | 13 (39.4%) |
| **Upper extremities motor score (out of 50 points)** |  |  |  |  |  |
| Mean ± SD | 39.9 ± 17.6 | 20.3 ± 25.7 | 26.0 ± 21.4 | 34.7 ± 14.4 | 34.0 ± 17.9 |
| Median [Min, Max] | 50.0 (0.00, 50.0) | 7.00 (4.00, 50.0) | 19.0 (9.00, 50.0) | 42.0 (12.0, 50.0) | 44.0 (0.00, 50.0) |
| Missing | 2 (16.7%) | 1 (25.0%) | 3 (50.0%) | 1 (9.1%) | 7 (21.2%) |
| **Lower extremities motor score (out of 50 points)** |  |  |  |  |  |
| Mean ± SD | 5.00 ± 15.8 | 5.00 ± 4.58 | 2.33 ± 3.21 | 46.2 ± 5.75 | 20.5 ± 23.1 |
| Median [Min, Max] | 0.00 (0.00, 50.0) | 6.00 (0.00, 9.00) | 1.00 (0.00, 6.00) | 49.5 (36.0, 50.0) | 6.00 (0.00, 50.0) |
| Missing | 2 (16.7%) | 1 (25.0%) | 3 (50.0%) | 1 (9.1%) | 7 (21.2%) |
| **Sensory score (touch) (out of 112 points)** |  |  |  |  |  |
| Mean ± SD | 48.4 ± 19.2 | 98.0 ± NA | 24.5 ± 6.36 | 89.8 ± 23.7 | 63.1 ± 31.3 |
| Median [Min, Max] | 46.5 (15.0, 74.0) | 98.0 (98.0, 98.0) | 24.5 (20.0, 29.0) | 96.5 (60.0, 112) | 60.0 (15.0, 112) |
| Missing | 4 (33.3%) | 3 (75.0%) | 4 (66.7%) | 5 (45.5%) | 16 (48.5%) |
| **Sensory score (pain) (out of 112 points)** |  |  |  |  |  |
| Mean ± SD) | 49.0 ± 19.1 | 98.0 ± NA | 24.0 ± 12.7 | 81.5 ± 22.7 | 60.4 ± 28.6 |
| Median [Min, Max] | 47.0 (15.0, 74.0) | 98.0 (98.0, 98.0) | 24.0 (15.0, 33.0) | 74.5 (61.0, 110) | 61.0 (15.0, 110) |
| Missing | 4 (33.3%) | 3 (75.0%) | 4 (66.7%) | 5 (45.5%) | 16 (48.5%) |
| **Time of ISNCSCI (days)** |  |  |  |  |  |
| Mean ± SD | 5.08 ± 2.68 | 4.00 ± 2.00 | 3.83 ± 0.983 | 4.73 ± 1.79 | 4.61 ± 2.06 |
| Median [Min, Max] | 4.00 (3.00, 10.0) | 3.00 (3.00, 7.00) | 3.50 (3.00, 5.00) | 5.00 (3.00, 7.00) | 4.00 (3.00, 10.0) |

To avoid the previously reported variability of the AIS grade during the first 2 d after SCI, we used the AIS grade assigned between days 3 and 10 after SCI in our analysis. Five out of 38 SCI patients whose blood was sequenced did not have an examination during that time period and were therefore excluded from the analysis.
