# Supplementary material for: Diagnostic blood RNA profiles for human acute spinal cord injury
Source: J Exp Med. 2021 Jan 29;218(3):e20201795. doi: 10.1084/jem.20201795 (PMC7852457; doi:10.1084/jem.20201795)
Supplement: Table S6 — shows the confusion matrix for the NLI predictive model. [file JEM_20201795_TableS6.docx]

Table S6. **Confusion matrix for the NLI predictive model**

|  | Reference | | |
| --- | --- | --- | --- |
| Prediction |  | Cervical | Other |
|  | Cervical | 16 | 5 |
|  | Other | 2 | 7 |

This table shows the performance of the model in predicting whether the SCI is cervical or not. Out of 18 cervical SCIs, the model correctly predicts 16 (sensitivity = 88.9%) but also misclassifies five as cervical (specificity = 58.3%). Overall, the model has 73.6% accuracy.
