# Supplementary material for: Diagnostic blood RNA profiles for human acute spinal cord injury
Source: J Exp Med. 2021 Jan 29;218(3):e20201795. doi: 10.1084/jem.20201795 (PMC7852457; doi:10.1084/jem.20201795)
Supplement: Table S7 — shows summary statistics of the NLI predictive model. [file JEM_20201795_TableS7.docx]

Table S7. **Summary statistics of NLI predictive model**

|  | Cervical |
| --- | --- |
| Accuracy | 0.7667 |
| 95% CI | (0.5772–0.9007) |
| No information rate | 0.6 |
| P value (accuracy > NIR) | 0.04352 |
| Sensitivity | 0.8889 |
| Specificity | 0.5833 |
| Positive predictive value | 0.7619 |
| Negative predictive value | 0.7778 |
| Prevalence | 0.6000 |
| Detection rate | 0.5333 |
| Detection prevalence | 0.7000 |
| Balanced accuracy | 0.7361 |
