# Supplementary material for: Diagnostic blood RNA profiles for human acute spinal cord injury
Source: J Exp Med. 2021 Jan 29;218(3):e20201795. doi: 10.1084/jem.20201795 (PMC7852457; doi:10.1084/jem.20201795)
Supplement: Table S8 — shows sequenced samples output and quality metrics. [file JEM_20201795_TableS8.docx]

Table S8. **Sequenced samples output and quality metrics**

|  | HC (*n* = 10) | TC (*n* = 10) | SCI (*n* = 38) | Overall (*n* = 58) |
| --- | --- | --- | --- | --- |
| Number of reads (in millions) |  |  |  |  |
| Mean ± SD | 45.7 ± 5.52 | 38.6 ± 5.10 | 41.0 ± 7.57 | 41.4 ± 7.13 |
| Median [Min, Max] | 45.9 (38.5, 56.6) | 38.9 (27.6, 44.5) | 40.5 (26.8, 73.4) | 40.9 (26.8, 73.4) |
| **% Perfect barcode** |  |  |  |  |
| Mean ± SD | 97.5 ± 0.593 | 96.7 ± 1.09 | 97.5 ± 0.696 | 97.4 ± 0.815 |
| Median [Min, Max] | 97.3 (96.8, 98.4) | 96.6 (95.2, 98.4) | 97.4 (96.1, 98.7) | 97.3 (95.2, 98.7) |
| **% One mismatch barcode** |  |  |  |  |
| Mean ± SD | 2.48 ± 0.593 | 3.33 ± 1.09 | 2.47 ± 0.696 | 2.62 ± 0.815 |
| Median [Min, Max] | 2.72 (1.60, 3.25) | 3.42 (1.59, 4.84) | 2.56 (1.28, 3.87) | 2.71 (1.28, 4.84) |
| **Yield (Mbases)** |  |  |  |  |
| Mean ± SD | 2,320 ± 288 | 1,940 ± 253 | 2,070 ± 387 | 2,090 ± 366 |
| Median [Min, Max] | 2,320 (1,950, 2,890) | 1,960 (1,410, 2,270) | 2,030 (1,340, 3,740) | 2,050 (1,340, 3,740) |
| **% ≥Q30 bases** |  |  |  |  |
| Mean ± SD | 97.5 ± 0.452 | 97.8 ± 0.226 | 97.7 ± 0.358 | 97.7 ± 0.362 |
| Median [Min, Max] | 97.6 (96.9, 98.0) | 97.9 (97.5, 98.0) | 98.0 (96.8, 98.1) | 97.9 (96.8, 98.1) |
| **Mean quality score (max = 40)** |  |  |  |  |
| Mean ± SD | 39.5 ± 0.133 | 39.6 ± 0.0529 | 39.6 ± 0.0992 | 39.6 ± 0.101 |
| Median [Min, Max] | 39.6 (39.4, 39.7) | 39.6 (39.5, 39.7) | 39.7 (39.3, 39.7) | 39.6 (39.3, 39.7) |
